# An evaluation of pathology foundation models with weakly supervised learning for BRAF mutation prediction in melanoma whole slide images

**DOI:** 10.3389/fmedt.2026.1835452

**Published:** 2026-09-03

**Authors:** Lucy Godson, Jack Breen, Alexander I. Wright, Derek Magee, Daljeet Bansal, Darren Treanor, Emily L. Clarke

**Affiliations:** 1 National Pathology Imaging Cooperative, Leeds Teaching Hospitals NHS Trust, Leeds, United Kingdom; 2 Division of Pathology and Data Analytics, University of Leeds, Leeds, United Kingdom; 3 Perspectum Ltd, Oxford, United Kingdom; 4 School of Computer Science, University of Leeds, Leeds, United Kingdom; 5 Department of Histopathology, Leeds Teaching Hospitals NHS Trust, Leeds, United Kingdom; 6 Center for Medical Image Science and Visualization, Linköping University, Linköping, Sweden

**Keywords:** BRAF prediction, computational pathology, melanoma, multiple instance learning, MIL, pathology foundation models

## Abstract

**Introduction:**

Melanoma is the most aggressive form of skin cancer, with around 40–50% of cases harbouring BRAF mutations that can be effectively targeted to improve patient outcomes. Typically, BRAF mutations are identified through DNA sequencing, which can be costly and contribute significantly to turn-around-times. Predicting BRAF status directly from haematoxylin and eosin (H&E) whole-slide images (WSIs) could offer a faster and more accessible alternative within digital pathology workflows.

**Methods:**

In this study, we evaluated six pathology foundation models for WSI-based BRAF mutation prediction using an attention-based multiple instance learning (ABMIL) framework. Training and testing models with five-fold cross-validation, using 1,109 WSIs from 745 patients and performing external validation with TCGA and VisioMel cohorts.

**Results:**

Models which utilised H-Optimus-1 features achieved the highest AUROC values on the internal test set (0.80 (0.72, 0.88)) and VisioMel (0.79 (0.75, 0.83) external test set, while UNI2-h features demonstrated superior sensitivity and higher TCGA performance (AUROC: 0.76 (0.66, 0.86)). Subgroup analyses indicated that model performance was impacted by histopathological features such as ulceration and tissue area, while t-SNE plots showed that high-attention foundation model features tended to cluster based on the dataset the features were from, rather than biological signal.

**Discussion:**

Overall, foundation models combined with weakly supervised learning provide competitive performance for BRAF status prediction in melanoma WSIs, but our findings highlight the need for diverse melanoma datasets and the importance of evaluating model performance beyond a single metric when developing H&E-based computational biomarkers.

## 1 Introduction

Melanoma is the fifth most common cancer in the UK, with a rising incidence (1). Mutations in the BRAF oncogene are found in approximately 40%–50% of melanomas. BRAF mutations are broadly divided into three functional classes: Class I mutations (most commonly V600 variants), which activate the MAPK signalling pathway driving uncontrolled cell growth and proliferation; Class II mutations (e.g., non-V600 mutations such as K601E), which also activate the MAPK signalling through alternative mechanisms; and Class III mutations (e.g., D594G) which are kinase-impaired and depend on upstream signalling alterations to become oncogenic. Accurately identifying these BRAF mutations is important for melanoma patient treatment decisions, for example BRAF inhibitors are a highly effective treatment against V600 BRAF-mutant melanoma, improving response rates, delaying disease progression, and extending overall survival in appropriately selected patients (2, 3). Currently, BRAF status is determined through DNA-based sequencing assays in advanced melanoma (4), but these tests can be costly and contribute significantly to turn-around-times. In addition, a recent large-scale study from the UK reported notable geographical and demographic disparities in access to BRAF testing (5). In settings where digital pathology is already established, automated prediction of BRAF mutation status directly from digitised haematoxylin and eosin (H&E) whole slide images (WSIs) offers a promising way to improve access to testing. Such image-based approaches could provide a rapid, cost-effective screening tool, helping clinicians identify which patients most urgently require confirmatory molecular testing. This may enable earlier treatment decisions, reduce turnaround times, and ultimately improve patient care.

Previous studies demonstrated that BRAF mutation status can be predicted from WSIs (6, 7), with BRAF mutant tumours exhibiting rounder and larger nuclear features. Kim et al. (6) reported a mean AUROC of 0.73 using an Inception v3 model trained on manually annotated regions from H&E-stained WSIs, whereas (7) achieved an AUROC of 0.63 when predicting BRAF mutations using features derived from H&E-stained WSIs using a random forest classifier. In more recent years, pathology foundation models paired with weakly supervised methods have demonstrated state-of-the-art performance for biomarker prediction (8–12), but performance for predicting BRAF status in melanoma WSIs has produced varying results with AUROC performance ranging from 0.57-0.87 (10, 11). Moreover these results are often reported on small, private or imbalanced test sets, without additional validation to determine if these models can generalize outside of the training distribution. A further challenge is the lack of attention to sensitivity-an essential metric when developing a screening tool intended to identify patients who should undergo confirmatory genetic testing for treatment decisions (13). Yet, successful clinical implementation requires rigorous evaluation beyond AUROC alone, including a detailed understanding of where and why models may fail. Without such comprehensive assessment, there is a risk that systems perform well under controlled benchmarking conditions but fail to deliver reliable, clinically meaningful predictions in real-world settings.

In this study, we perform a thorough evaluation of six current state-of-the-art pathology foundation models, with attention based multiple instance learning (ABMIL) frameworks for BRAF status prediction from WSIs. Our results confirm that pathology foundation models with ABMIL can achieve strong performance for mutation classification, and, to our knowledge, this is the first work to exceed an AUROC of 0.7 on two publicly available external datasets for predicting BRAF status from WSIs (VisioMel: AUROC 0.791 [95% CI: 0.754–0.828], TCGA: 0.787 [95% CI: 0.682–0.885]). Furthermore, the inclusion of multiple metrics reveals substantial variability in sensitivity on external validation sets (0.095–0.810), which is strongly influenced by the choice of pathology foundation model used for feature extraction. In addition, through pathologist-guided interpretation of attention heatmaps, we show that rare melanoma subtypes and variations in staining or slide quality can adversely affect model predictions. By examining the foundation model feature space learned by each ABMIL model, we also show that many models rely on dataset-specific rather than biologically relevant features, which could lead to diagnostic errors. Finally, we perform exploratory subgroup analyses, which to our knowledge have not been previously used for BRAF status classification in melanoma WSIs. Here, we examine whether weakly supervised model classification performance is impacted by histopathological characteristics such as histolologic subtype, ulceration and tissue area. This underscores the need for more robust and biologically grounded approaches to computational biomarker development.

## 2 Materials and methods

### 2.1 Patch encoders and model training

For feature extraction we used the TRIDENT codebase (14, 15) to implement six different foundation models: H-Optimus-1 (16), Phikon-v2 (17), UNI2-h (18), CONCHv1.5 (19), MUSK (20) and Virchow2 (12) (shown in Table 1). These models were chosen as H-Optimus-1 (16), UNI2-h (18) and Virchow2 (12) have shown superior performance across multiple pathology benchmarking tasks (21), Phikon-v2 has shown competitive performance for biomarker prediction (17), CONCHv1.5 has been shown to be robust against learning non-biological artifacts across different datasets (22) and MUSK has been shown to perform well on the VisioMel melanoma dataset for relapse prediction (20).

**Table 1:** Foundation model patch encoders with the differing architectures, number of parameters (in millions), input patch sizes and embedding sizes generated from the model.

Patch encoder	Architecture	#Parameters	#Pretraining WSIs	Patch size	Embedding dim
Phikon-v2	ViT-Large	303 M	58.4 K	224	1,024
CONCHv1.5	ViT-Large	307 M	100 K${}^{a}$	512	768
Virchow2	ViT-Huge	632 M	3.1 M	224	2,560
MUSK	BEiT3	675 M	33 K${}^{a}$	384	1,024
UNI2-h	ViT-Huge	681 M	350 K	256	1,536
H-Optimus-1	ViT-Giant	1,100 M	>1 M	224	1,536

${}^{a}$Indicates vision language models which were additionally trained with image and text pairs.

Before patch extraction, tissue was segmented from the background of each WSI using a segmentation model based on DeepLabV3 pretrained on the COCO dataset (15, 23). Then to generate feature embeddings, we chose to use the published default tiling parameters (including patch resolution) for each foundation model, as these were likely to be the parameters which led to optimal performance for each model. This meant extracting non-overlapping tiles at 20× resolution and patch sizes ranging from 224×224 to 512×512 pixels depending on the foundation model. The patch sizes and the output embedding sizes generated are recorded in Table 1.

To aggregate the patch embeddings, ABMIL models (24) were implemented and trained using the Patho-bench codebase (14, 15). Embeddings were aggregated at a patient-level during training and testing, to generate **z**, a patient-level representation, which is the aggregation of the patch feature embeddings and corresponding weights:

$$z=\sum_{k=1}^{K}a_{k}h_{k},$$

where $h_{k}$ is the $k^{th}$ patch instance embedding and $a_{k}$ is the weight that is derived from the neural network’s attention backbone. The attention weights ($a_{k}$) sum to one to be invariant of the number of patch embeddings in a slide and ensure that the weights are interpretable as relative contributions to the overall patient-level prediction. This means that the instances ($h_{k}$) with higher attention weights ($a_{k}$) contribute more to the aggregated patient-level representation (**z**). The weights for the attention mechanism are formulated by:

$$a_{k}=\frac{\exp\;\left(w^{T}\tanh\;({Vh}_{k}^{T})\right)}{\sum_{\;j=1}^{K}\exp\;\left(w^{T}\tanh\;({Vh}_{j}^{T})\right)},$$

where $w\in R^{d\times 512}$ and $V\in R^{512\times 256}$ are the weights of the first and second fully connected layers of the neural network, respectively and d represents the dimensionality of the input feature embeddings, which differ according to the embedding size of the foundation model (Table 1). During each training epoch 2,048 patch features were randomly sampled from each image, to reduce training time and can help to reduce overfitting.

ABMIL models were trained for 20 epochs using five-fold cross-validation with patient-level splits of 60% train, 20% validation, and 20% test, using the Leeds Melanoma cohort (LMC) WSIs. A base learning rate of 0.0003 was applied with AdamW optimiser, cosine annealing and weight decay of 0.00001. Validation loss was monitored throughout training and the model checkpoint with the lowest validation loss was saved. This saved model was then used to test the internal hold-out test set and two external test sets. Performance was based on the ensembled mean result across the five models and 95% confidence intervals were estimated via 1,000 replicates of bootstrapping. We report AUROC, sensitivity and specificity for all ABMIL classifiers. The decision threshold was fixed at 0.5 for all models when computing sensitivity and specificity and this was applied to external test sets. DeLong tests (25) were implemented to determine whether differences in AUROC values were statistically significant and p-values were corrected for multiple comparisons using the Holm–Bonferroni method (26). We also performed ranked Wilcoxon signed-rank tests (27) to compare the sensitivity and then specificity values for paired models being compared across the five folds. With only one value per fold and a sample size of *n* = 5, these results were not significant, but are included in the Supplementary Section with a full report of the DeLong tests across the three test sets.

The code used to perform feature extraction using the foundation models and train the ABMIL models was accessed from the following repositories https://github.com/mahmoodlab/TRIDENT and https://github.com/mahmoodlab/Patho-Bench (last accessed: March 2026).

### 2.2 Visual attention maps

Visual attention maps were developed by using the attention weight scores ($a_{k}$) for the patch embeddings. The attention scores were then scaled with the highest (1.0), being the most highly attended patch for the predicted slide-level label and the lowest (0.0) being the least attended patch for the predicted slide-level label. The scores were then converted to a red-blue colour map which is overlaid on the WSI, with red tiles indicating highly attended patches and blue tiles indicating low attention patches, which contribute less to the BRAF status prediction. Slides were selected from the test set for heatmap generation if the majority of models correctly classified the case with high agreement, or if all models consistently misclassified it.

### 2.3 t-SNE feature space plots

To explore the feature space of the learned high attention embeddings from different foundation models we generated separate t-SNE plots. PCA was used to extract the top 30 components from the top 20 high attention features learned from test set images across the three datasets. The t-SNE projections were implemented using the sklearn package https://scikit-learn.org/stable/modules/generated/sklearn.manifold.TSNE.html, with 2 components, perplexity of 140, learning rate of 800, PCA initialization and early exaggeration of 50.

### 2.4 Exploratory subgroup analysis

In assessing whether different models exhibited systematic performance dispartites across patient subgroups, we conducted a series of exploratory subgroup analyses based clinically relevant demographic and pathological characteristics. Patients were stratified by age (<50 years vs. ≥50 years), sex (male or female), presence of ulceration (presence, absence or unknown), Breslow thickness (<1.0 mm, [1.0–2.0 mm), [2.0–4.0 mm), and ≥4.0 mm), histological subtype (Superficial Spreading Melanoma [SSM], Nodular Melanoma [NM], Lentigo Malignant Melanoma [LMM], Acral Lentiginous Melanoma [ALM], other, or unclassified), and the anatomic site of the primary tumour (trunk, non-trunk, or unknown). These characteristics were selected both for their clinical relevance and because they are associated with BRAF mutation status (28–32); thus, examining performance across these subgroups allowed us to assess whether model predictions aligned with established predictors of BRAF status.

For one model, we additionally evaluated the potential influence of tissue area by examining the number of patch embeddings per whole-slide image. After segmentation to remove background, slides were grouped into five bins based on their total number of tissue patches.

For each subgroup, we calculated sensitivity, specificity, and AUROC to quantify model performance. There is a detailed breakdown of the clinical and pathological characteristics of the datasets in the Supplementary Material.

### 2.5 Datasets

To train, validate and test the ABMIL models we used 1,109 slides from 745 patients (44.6% BRAF-mutant) from the Leeds Melanoma Cohort (LMC), a population ascertained cohort from the North of England (33, 34). These slides come from Formalin-Fixed Paraffin-Embedded (FFPE) blocks and were scanned in batches using a Leica Bisosystems Aperio Digital Pathology Slide Scanner, at 0.25 μm/pixel. Ground truth BRAF status for each WSI was obtained from routine clinical molecular testing, including pyrosequencing, next-generation sequencing and real-time PCR (Polymerase Chain Reaction) assays such as the Cobas 4,800 system, CE-SSCA (Capillary Electrophoresis—Single-Strand Conformation Analysis), and Sanger sequencing, depending on the hospital laboratory performing the test. Tumours were classified as BRAF-mutant if any pathogenic BRAF variant was reported. The BRAF-mutant class comprised V600E mutations (78%), V600K (14%), V600E mutations detected in a deletion–insertion (delins) form (2%), K601E (2%), V600D (1%), and unspecified or other complex BRAF mutations (3%). Cases in which no BRAF mutation was detected by the clinical assay were labelled BRAF wild-type for the corresponding WSIs.

A total of 523 WSIs from the VisioMel Challenge dataset, comprising melanoma WSIs curated from the French national collection, were selected as an external validation test set, as they had been assessed for V600E BRAF mutations (35). For each case, a pathologist chose a single representative slide, and all slides were digitised using various scanners at an approximate resolution of 0.25 μm/pixel. Overall, 40.7% of the slides were classified as BRAF-mutant. The dataset was obtained from the VisioMel database: https://www.data.gouv.fr/datasets/visiomel-database-predicting-cutaneous-melanoma-relapse [last accessed March 2026].

In addition, we ultilised WSIs from the TCGA-SKCM dataset (36). This dataset contains 475 slide-labeled FFPE H&E slides, but we selected only cases with a primary tumour diagnosis (*n* = 168), as the LMC dataset contained only primary melanomas and previous studies have shown that performance of molecular prediction models can be reduced on metastatic specimens (13). In addition we further restricted the WSIs we used to those with a primary site of the skin (*n* = 88), as some WSIs which were included as primaries had a tumour site lymph nodes and connective, subcutaneous and other soft tissues, which are extremely rare for primary melanomas. Moreover, an expert pathologist (EC) examined the TCGA WSIs and found some cases which were thought to be melanoma primaries from our initial TCGA database search, but were likely to be metastases (*n* = 8) or were a different type of skin cancer (*n* = 1) or not skin cancer (*n* = 1), so we removed these WSIs, leaving 83 slides from 78 patients (53.8% with BRAF mutated tumours). Out of the BRAF-mutated tumours 91% had V600E mutations and 9% were other mutations such as K601E and V600G. 82 slides were scanned at 0.25 μm/pixel and 1 slide was digistised at 0.5 μm/pixel. The diagnostic digital slides were downloaded using the GDC Data Transfer Tool: https://portal.gdc.cancer.gov [last accessed March 2026].

## 3 Results

### 3.1 Comparison of model performance

**Figure 1:**
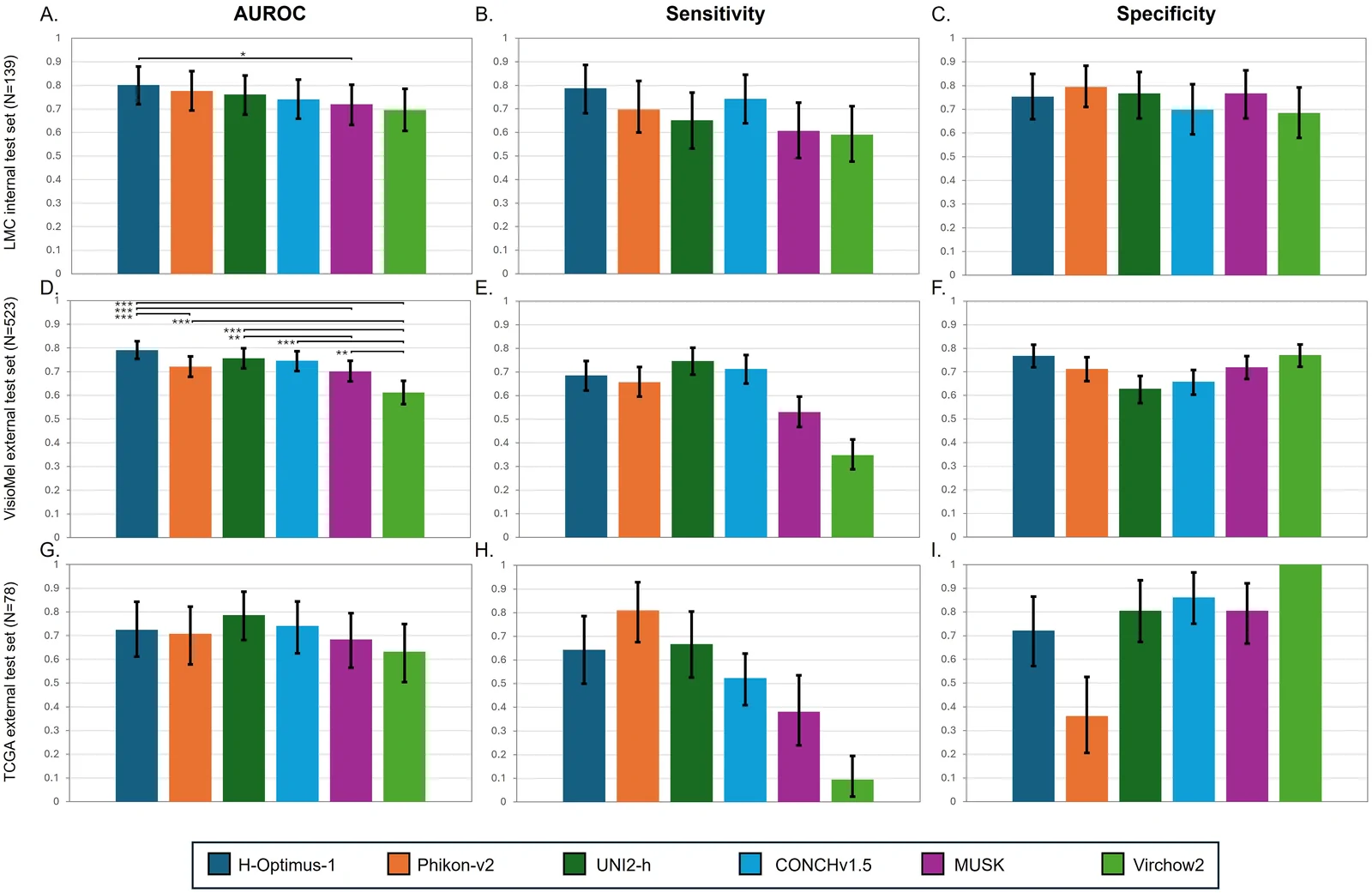
Performance of models for predicting BRAF status. Mean test performance with 95% confidence intervals is shown for each dataset. AUROC, sensitivity, and specificity are reported for attention-based multiple instance learning models, evaluated using features extracted from different foundation models. Results are shown for the LMC cohort **(A–C)**, the VisioMel external cohort **(D–F)**, and the TCGA external test set **(G–I)**. Phikon-v2 was reported to have been pretrained on TCGA images, which should be considered when interpreting its performance on that dataset. Adjusted p-values from DeLong tests are shown, where * indicates P<0.05, ** indicates P<0.01, *** indicates P<0.001.

ABMIL models trained with H-Optimus-1 features achieved the highest AUROC on both the internal LMC test set (Figure 1A; 0.801 [95% CI 0.720–0.880]) and the VisioMel external validation set (Figure 1D; 0.791 [95% CI 0.754–0.828]). When evaluated on the VisioMel dataset, the H-Optimus-1-based model significantly outperformed models based on Phikon-v2 (AUROC 0.721, $P=1.01\times 10^{-4}$), MUSK (AUROC 0.701, $P=6.30\times 10^{-6}$), and Virchow2 features (AUROC 0.612, $P=5.63\times 10^{-14}$), with AUROC improvements of +0.070, +0.080 and +0.145 respectively. On the smaller internal LMC test set, the H-Optimus model also achieved a significantly higher AUROC compared to the MUSK-based model (AUROC 0.719, P=0.0364) with improvements ranging from +0.025 to +0.105 over the other models.

In contrast, on the TCGA dataset, the UNI2-h-based model achieved the highest AUROC (0.763 [95% CI 0.656–0.862]), exceeding the H-Optimus-1-based ABMIL model (0.701 [95% CI 0.581–0.804]) by +0.0615. The H-Optimus-1 model, in turn, performed 0.0218 lower than the AUROC of the ABMIL model trained with CONCHv1.5 features (AUROC = 0.728 [95% CI 0.611–0.832]; Figure 1G). However, confidence intervals were wider for the smaller TCGA external validation set (Figures 1G–I), and adjusted p-values from DeLong tests were not significant (Supplementary Table S3), limiting confidence in these apparent differences. Consistent with this, no statistically significant differences in AUROC were observed between UNI2-h, CONCHv1.5, and H-Optimus-1-based models across the VisioMel and LMC test sets. Notably, these models vary considerably in size [CONCHv1.5: 307M parameters; UNI2-h: 681M; H-Optimus-1: 1100M (Table 1)], yet achieve broadly comparable performance, suggesting that larger model capacity does not necessarily confer a performance advantage in this task. Furthermore, when evaluated on the VisioMel dataset, the UNI2-h model achieved a significantly higher AUROC than MUSK and Virchow2 models [$P=7.24\times 10^{-3}$ and $P=4.30\times 10^{-8}$, respectively (Figure 1)], despite similar parameter counts [UNI2-h: 681M; MUSK: 675M; Virchow2: 632M (Table 1)], further indicating that model size alone does not explain performance differences.

One possible explanation for these small performance differences is geographical bias, as suggested by (37). The TCGA dataset is derived from North American institutions, and both UNI2-h and CONCHv1.5 use training data from North America (e.g., H&E and immunohistochemistry slides from Mass General Brigham). In contrast, H-Optimus-1 was developed by a French company (Bioptimus) and may have been trained on data more similar to the French VisioMel cohort, which could partly explain its relatively strong performance on this dataset. However, as details of the training data are not fully disclosed, this interpretation remains speculative.

When considering class-specific performance, the ABMIL models trained on UNI2-h features outperform those trained on H-Optimus-1 features across both external validation sets (VisioMel: 0.7465 [95% CI 0.688–0.803] vs. 0.685 [95% CI 0.621–0.746]; TCGA: 0.667 [95% CI 0.526–0.805] vs. 0.643 [95% CI 0.500–0.786]), suggesting improved detection of BRAF-mutant cases. Phikon-v2, which was trained on publicly available data including the TCGA dataset, achieved the highest sensitivity on the TCGA set (Figure 1H), but at the cost of reduced specificity (0.3611 [95% CI 0.2059–0.5263]), indicating poorer perfomance at classifying BRAF wild-type cases. In contrast, models trained with MUSK and Virchow2 features demonstrated consistently high specificity across external test sets (0.719–1.0, shown in Figures 1F,I), but with low sensitivity values (0.095–0.530, shown in Figures 1H,E), suggesting a bias towards predicting the majority BRAF wild-type class. Notably, Virchow2-based models also produced significantly lower AUROC values than all other models on the VisioMel dataset (P<0.005; Figure 1D).

### 3.2 Attention heatmaps from ABMIL models

**Figure 2:**
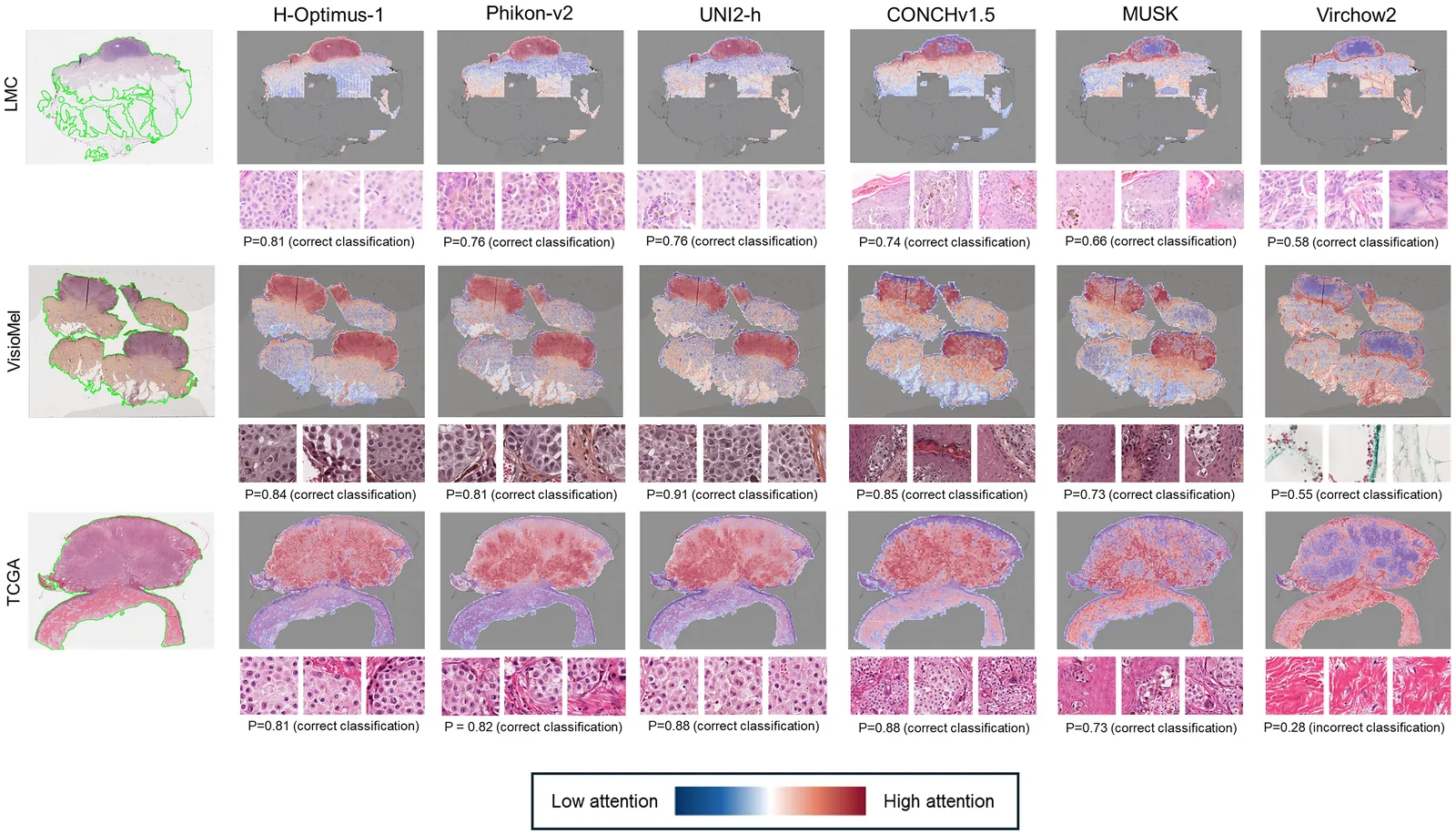
Comparison of attention heatmaps from ABMIL models when classifying BRAF-mutant cases across three independent datasets. Each row shows a H&E thumbnail with the corresponding attention heatmap overlay, top-3 high attention patches and the prediction score for the BRAF-mutant test set case. The columns show the results from the six different foundation models used for feature extraction (H-Optimus-1, Phikon-v2, UNI2-h, CONCHv1.5, MUSK, Virchow2).

**Figure 3:**
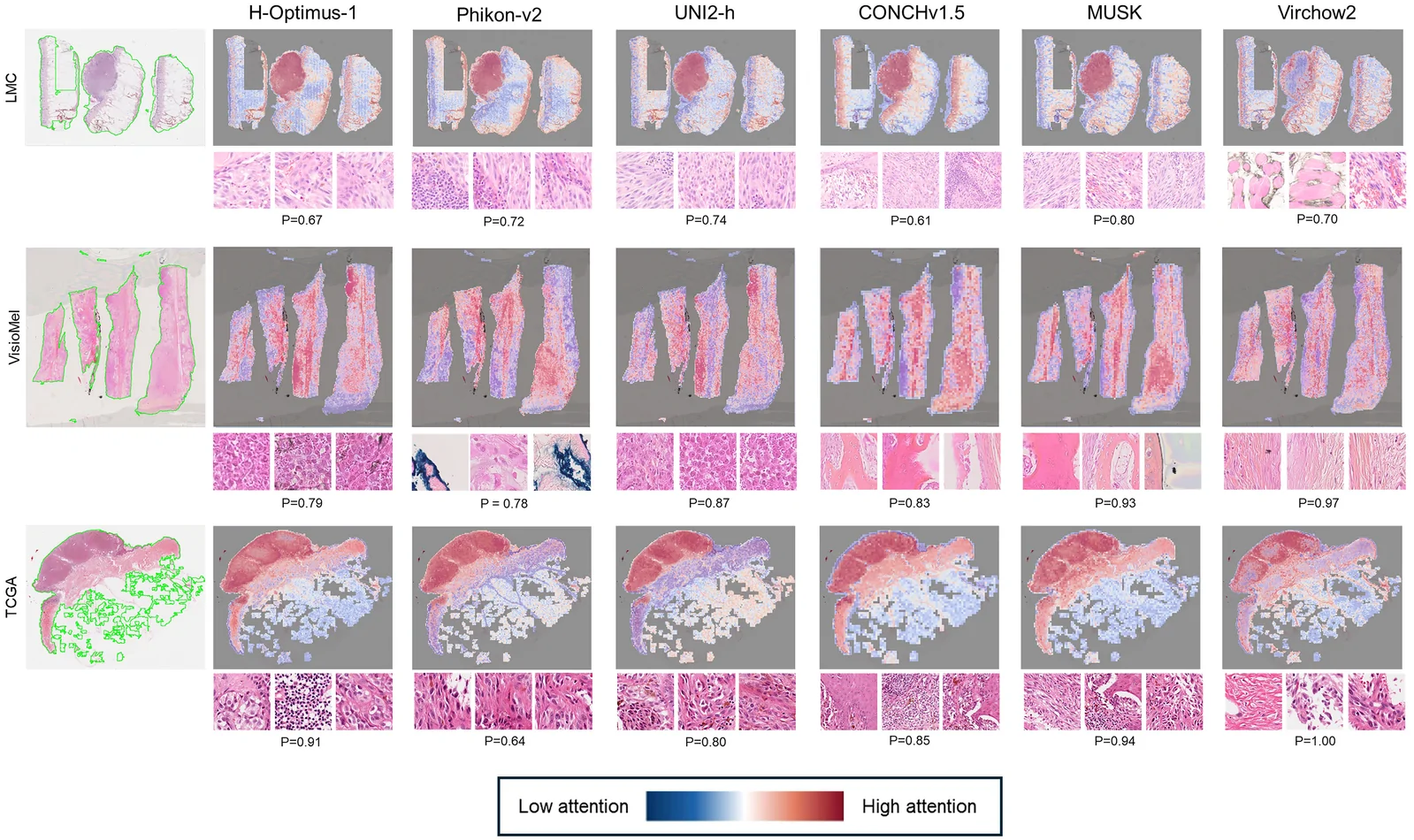
BRAF-mutant melanoma cases incorrectly classified as BRAF wild-type. Each row contains a H&E thumbnail with the corresponding attention heatmap overlay, top-3 high attention patches and the prediction score for the misclassified test set cases. The columns show the results from the six different foundation models used for feature extraction (H-Optimus-1, Phikon-v2, UNI2-h, CONCHv1.5, MUSK, Virchow2).

In Figures 2, 3, we present attention heatmaps for BRAF mutant cases that were correctly and incorrectly classified across the three datasets. Attention heatmaps and high attention patches can provide useful exploratory insights into model behaviour, however, they do not constitute definitive evidence that models rely on BRAF associated morphological features (38). Inspection of high attention regions suggests consistent qualitative patterns across models. For correctly classified cases, models trained with H-Optimus-1, UNI2-h and Phikon-v2 tended to assign higher attention scores to similar regions (Figure 2). These regions frequently corresponded to tumour areas enriched with cells exhibiting enlarged nuclei. In contrast, CONCHv1.5 and MUSK features are extracted from larger patch sizes (512×512 and 384×384 pixels, respectively), incorporating a broader spatial context. Their high attention regions more frequently extend beyond dense tumour areas, often including surrounding tissue (Figures 2, 3). Although additional context can be beneficial for certain tasks, such as immune subtyping (39), these observations suggest that, for BRAF status prediction, models focusing more narrowly on tumour rich regions may achieve improved performance. Larger contextual fields may introduce additional variability or noise, particularly in melanoma, which is characterised by substantial intra-tumoural heterogeneity.

Despite also using 224×224 pixel inputs, models trained with Virchow2 features frequently assign higher attention to non tumour regions and are more often associated with incorrect or low confidence predictions (Figure 1H). This is notable given prior reports of strong Virchow2 performance across diverse histopathology tasks (11, 21). Virchow2 was pretrained on a large and diverse dataset (3.1 million WSIs, Table 1), including approximately 10% skin tissue (12), although the representation of melanoma specifically is unclear. Melanoma exhibits substantial morphological heterogeneity compared to other skin conditions, which may limit the transferability of pretrained features. Moreover, previous work reporting high performance for BRAF classification (AUROC 0.87; sensitivity 0.95) was based on a relatively small and imbalanced dataset (n = 121; 16% BRAF mutant) and showed poor specificity (0.30). Taken together with the low sensitivity observed in our study, these findings suggest that Virchow2 features may have limited discriminative ability for BRAF mutation status in melanoma without additional fine-tuning.

For the BRAF-mutant misclassified cases in Figure 3, all models fail to correctly classify the LMC BRAF-mutant case, which contains rare spindle-cell morphology. Instead of highlighting the regions with round nuclei contained within the tumour, many models assign high attention to elongated spindle-cell areas, suggesting models are unable to generalise to tumours that contain rare cell variants. In addition the VisioMel misclassified case in Figure 3, illustrates how staining variation and blur could be impacting model performance. The reviewing pathologist (EC) noted that this image had insufficient haematoxylin, poor nuclear visibility, and a yellow hue consistent with Haematoxylin–Eosin–Saffron staining, commonly used in France for collagen. This variation in staining and image quality may have contributed to all models misclassifying this case.

### 3.3 Qualitative assessment of unsupervised BRAF features

**Figure 4:**
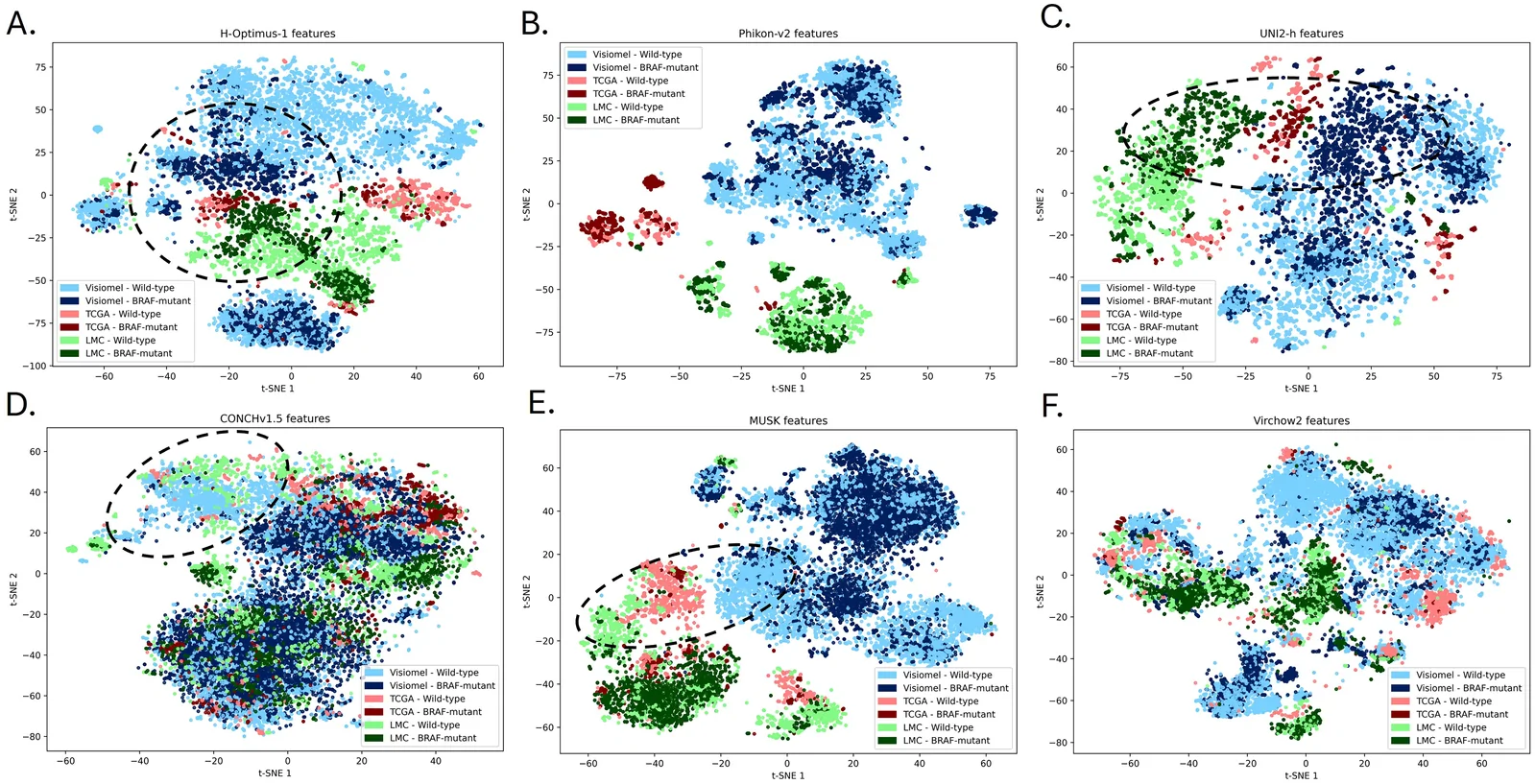
T-SNE projections of the top 20 high attention features learned by the ABMIL models for each WSI from the three test datasets across the six foundation models [**(A)**, H-Optimus-1, **(B)**, Phikon-v2, **(C)**, UNI2-h, **(D)**, CONCHv1.5, **(E)**, MUSK and **(F)**, Virchow2]. Regions that contain high attention features from the same class, but from different datasets have been circled with a dashed ellipse.

T-SNE feature space plots of H-Optimus-1 and UNI2-h features show clustering of BRAF mutant embeddings in feature space (circled in Figures 4A,C). This pattern is consistent with their higher sensitivity across datasets, as they have a stronger discrinative ability for BRAF mutant cases. In contrast the plots showing Phikon-v2, CONCHv1.5, MUSK, and Virchow2 features lack comparable BRAF-positive clusters. In comparison, CONCHv1.5 and MUSK features show some tighter clustering of BRAF-negative features (circled in Figures 4D,E). However, more broadly, most of the high attention features from the foundation model patch encoders tend to cluster according to dataset provenance rather than BRAF status (Figure 4). This observation is consistent with prior reports that foundation models can be sensitive to technical variation, such as scanner type, staining protocols, or site-specific characteristics (22, 40). Such dataset-dependent structure in feature space may influence downstream model behaviour and could contribute to reduced generalisability.

This effect appears most pronounced for Phikon-v2 features (Figure 4B), where high-attention regions show clear clustering by dataset. This pattern suggests that these representations may be influenced by dataset-specific factors, rather than capturing signals that generalise consistently across cohorts. CONCHv1.5 features appear more interspersed overall—aside from a distinct BRAF-negative cluster (circled in Figure 4D), indicating a more heterogeneous feature distribution. This may reflect reduced sensitivity to dataset-specific structure, consistent with previous observations (22, 40). Such heterogeneity could arise from the larger patch size used for CONCHv1.5 feature extraction, which incorporates a broader range of tissue and cellular context, or from the vision–language pretraining of CONCHv1.5 with diverse multimodal data.

### 3.4 Subgroup performance based on clinical and histopathological features

**Figure 5:**
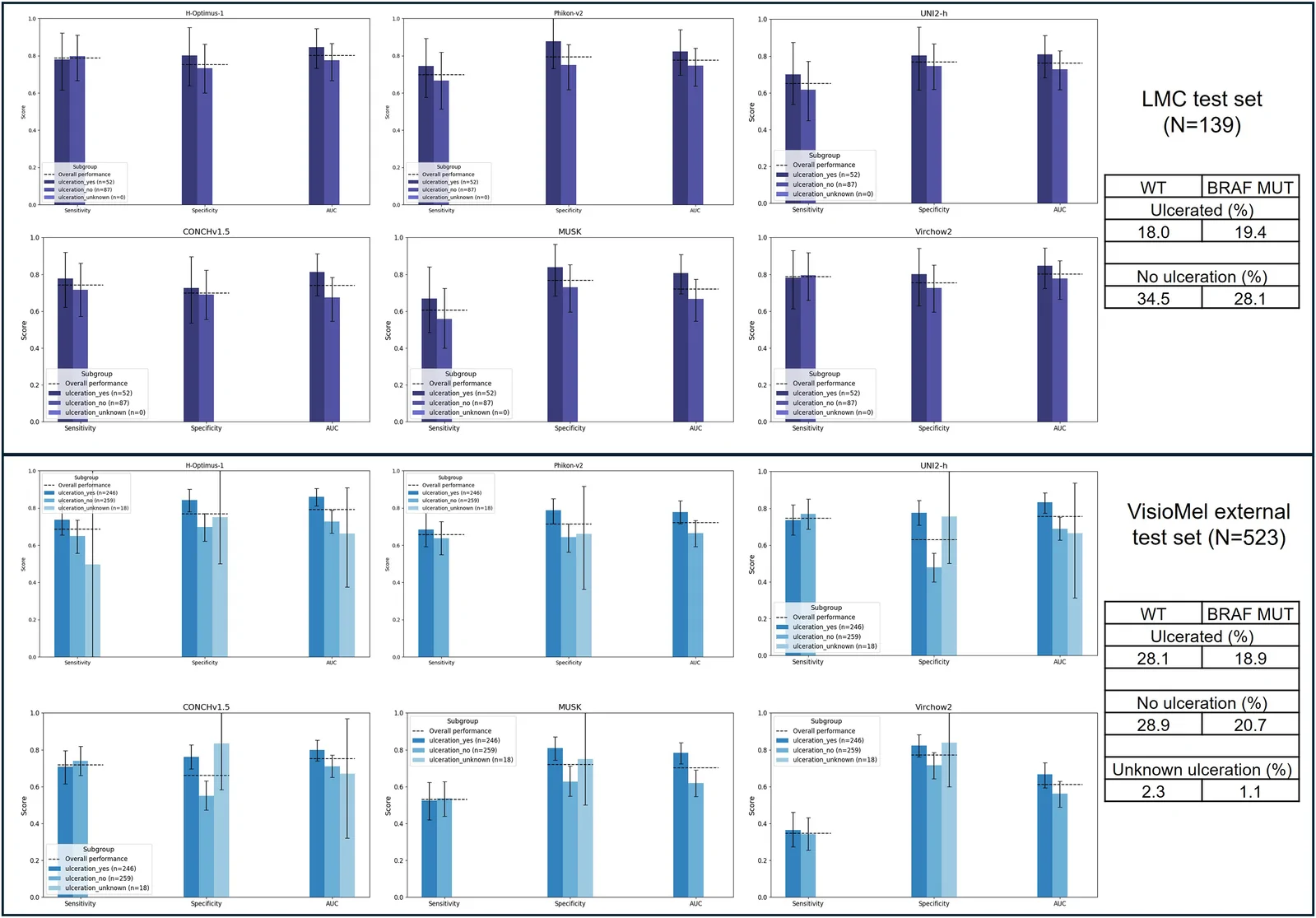
Performance gaps between patient subgroups with ulcerated vs. non-ulcerated tumours. Each plot is from a model trained with different foundation model features and shows mean sensitivity, specificity and AUROC values with 95% CI. The first panel shows results from the LMC test set the second panel shows results from the VisioMel test set. The dashed line indicates the overall sensitivity, specificity and AUROC for each model on the entire test set. The tables show the proportion of patients, which have wild-type (WT) or BRAF-mutant (BRAF MUT) tumours for the different ulceration categories within the LMC and VisioMel test sets.

**Figure 6:**
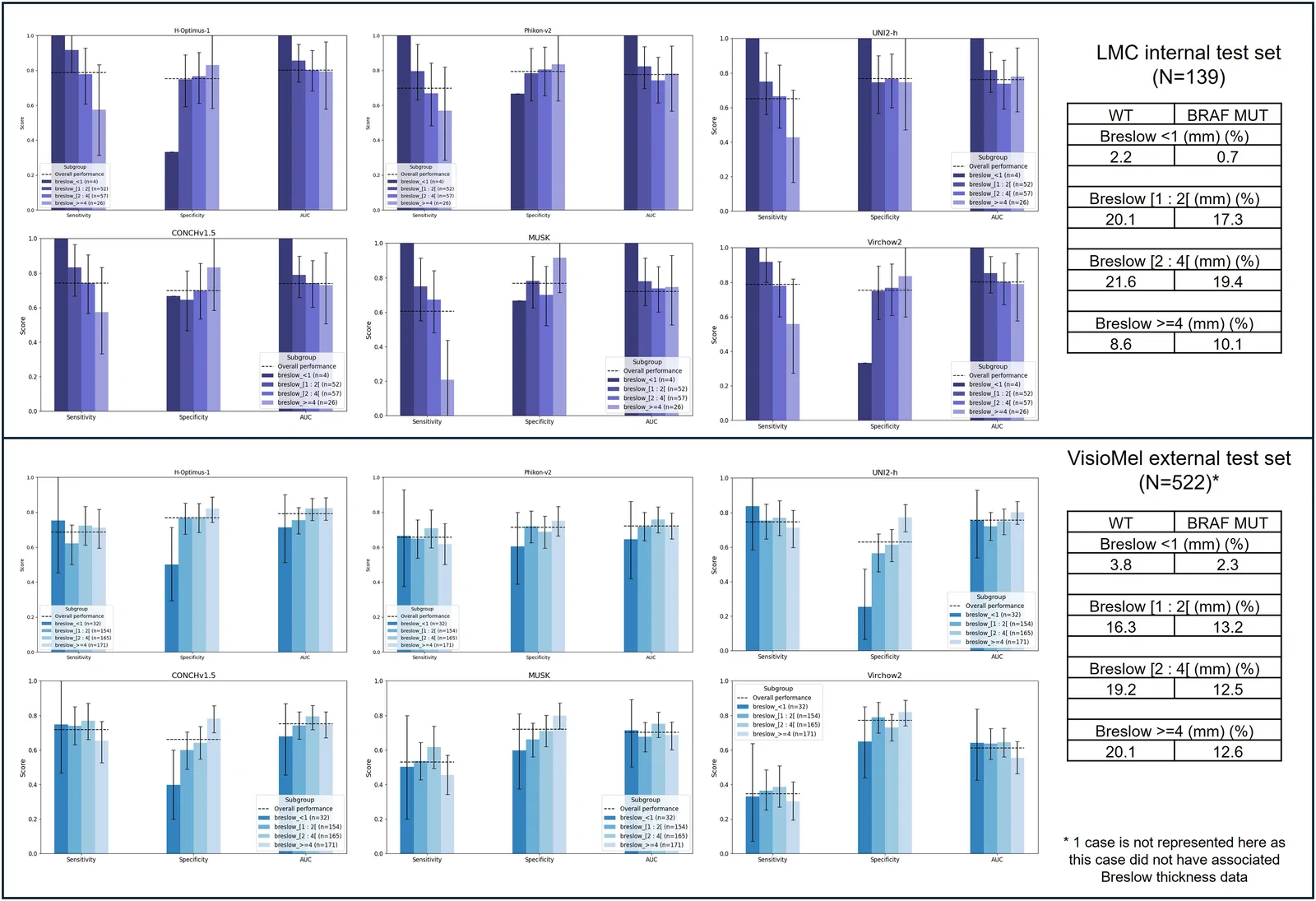
Performance gaps between patient subgroups with differing Breslow thickness of tumours. Each plot is from an ABMIL model trained with different foundation model features and shows the mean sensitivity, specificity and AUROC values with 95% CI. The first panel shows results from the LMC test set and the second panel shows results from the VisioMel test set. The dashed line indicates the overall sensitivity, specificity and AUROC for each model on the entire test set. The tables show the proportion of patients which have wild-type (WT) or BRAF-mutant (BRAF MUT) tumours for the different Breslow thickness categories within the LMC and VisioMel test sets.

In addition to exploring attention heatmaps and feature spaces learned by ABMIL models trained with different foundation-model features, we performed an exploratory evaluation of model performance across clinically relevant subgroups. We focused on the LMC test set and the VisioMel external validation dataset, as the TCGA dataset contained a large proportion of missing or unclassified pathological data (Supplementary Table S10). As shown in Figure 5, all models, irrespective of the foundation model used for feature extraction, demonstrated higher AUROC when classifying tumours with ulceration compared to non-ulcerated tumours. In the LMC test set, AUROC values were higher by 0.065–0.140 in ulcerated tumours. This pattern was replicated in the larger VisioMel external validation test set, where AUROC values were 0.092–0.166 higher in ulcerated tumours compared to non-ulcerated tumours. Ulceration is a histopathological feature characterised by breakdown of the epidermis overlying the melanoma and is incorporated into staging due to its association with more aggressive tumour behaviour and poorer prognosis (41). Improved model performance for ulcerated tumours may suggest that classifiers are leveraging morphological features associated with ulceration to support prediction. However, because ulceration is also associated with increased tumour thickness, we also assessed performance across subgroups defined by Breslow thickness, a key measure of melanoma depth (Figure 6).

In Figure 6, interpretation of the performance of the models on thin melanomas (<1 mm) for the LMC test set is challenging due to the very small number of cases (n = 4), resulting in unstable estimates. The 95% CI are also very wide for thinner tumours in the VisioMel test set. Among thicker tumours (≥1 mm) in the LMC test set, we observed a trend towards increasing specificity with increasing thickness, while sensitivity tended to decrease, and AUROC did not appear to improve. A similar pattern with increased specificity performance, as Breslow thickness increased was observed in the VisioMel dataset for several models (H-Optimus-1, UNI2-h, CONCHv1.5, and MUSK), which may indicate a bias towards predicting thicker tumours as BRAF wild-type. However, given the wide confidence intervals and small subgroup sizes, these observations should be interpreted cautiously.

While tumour thickness may influence model performance, (13) previously reported improved mutation-classification performance with increasing amounts of available tissue in whole-slide images. To further investigate whether observed performance differences were driven by tumour thickness or by the amount of tissue present, we evaluated performance in the VisioMel test set after stratifying slides by the number of extracted patches (used as a proxy for tissue area) (Table 2). Dividing the dataset into five groups, we found that the subgroup with the second-highest number of patches (patch range [19,070, 26,771], Table 2) achieved the highest AUROC across all models. Notably, this subgroup had similar proportions of tumours >2 mm and comparable rates of ulceration relative to the subgroup with the greatest number of patches (as shown for H-Optimus-1 in Table 3), yet showed substantially better performance (AUROC 0.883 vs. 0.756; sensitivity 0.756 vs. 0.698; specificity 0.814 vs. 0.726). In contrast, the subgroup containing slides with the lowest number of extracted patches (patch range [380, 7,432]) showed the lowest sensitivity (0.543) but the highest specificity (0.829).

**Table 2:** Analysis of model performance stratified by the number of patches or features per slide for the ABMIL model trained with H-Optimus-1 features.

Patch range	AUC	Sensitivity	Specificity	Median patches	Slides	BRAF-mutant
[380, 7,432]	0.751	0.543	0.829	4,187	105	35
[7455, 14,258]	0.768	0.702	0.719	11,295	104	47
[14272, 19,063]	0.746	0.698	0.742	16,314	105	43
[19070, 26,771]	0.883	0.756	0.814	22,183	104	45
[26913, 105,792]	0.790	0.698	0.726	33,370	105	43

The last two columns show the number of slides in each range and the number of BRAF-mutant slides. The results shown are from the VisioMel dataset.

**Table 3:** The number of slides with different ulceration and Breslow characteristics across the different patch-count ranges. The results shown are from the VisioMel dataset for the ABMIL model trained with H-Optimus-1 features.

Patch range	Ulceration	Breslow 1–2 mm	Breslow 2–4 mm	Breslow ≥4 mm
[380, 7,432]	37	51	28	16
[7455, 14,258]	38	39	36	22
[14272, 19,063]	51	23	44	33
[19070, 26,771]	59	19	31	49
[26913, 105,792]	61	22	26	51

The results shown are from the VisioMel dataset.

Overall these findings suggest that factors beyond tumour thickness or ulceration could be contributing to improved model performance. Specifically, there may be an optimal range of tissue quantity, morphological diversity, or contextual information that supports more accurate classification, rather than a simple monotonic relationship with tumour size or area. Alternatively, slides with the largest tissue areas may include a greater proportion of non-informative regions alongside tumour tissue, potentially diluting relevant signal. Notwithstanding this, the low sensitivity of the cases with the lowest patch range suggest that detection of BRAF-mutant tumours may be more challenging in thinner tumours (<1 mm and 1–2 mm) or slides with limited tissue representation. However, this observation is difficult to confirm given the small number of tumours < 1 mm represented in the LMC and VisioMel datasets.

**Figure 7:**
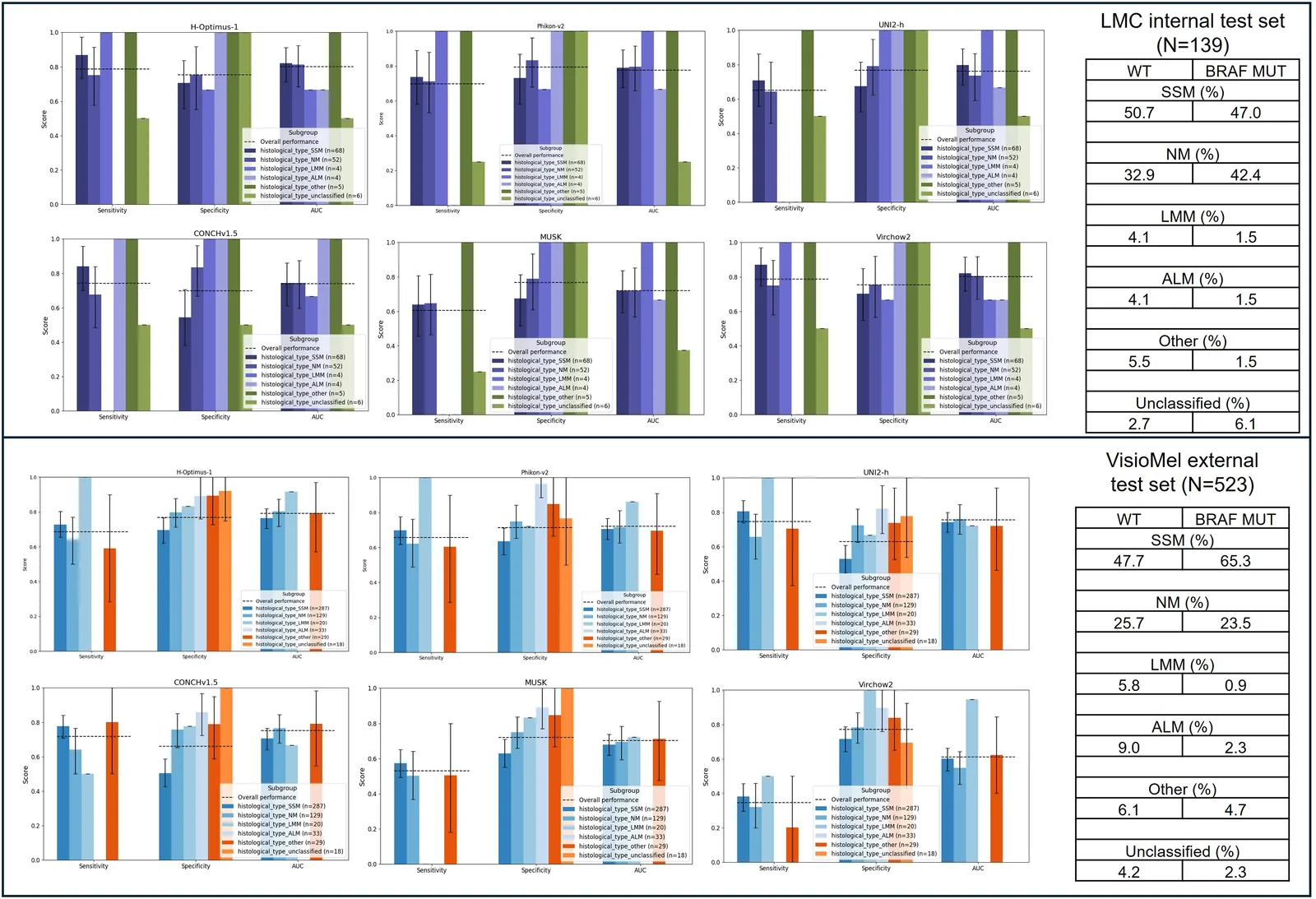
Performance gaps between patients with melanomas which are different histological subtypes. Each plot is from a model trained with different foundation model features and shows mean Sensitivity, specificity and AUROC values with 95% CI. The first panel shows results from the LMC test set the second panel shows results from the VisioMel test set. The dashed line indicates the overall sensitivity, specificity and AUROC for each model on the entire test set. The tables show the proportion of patients which have wild-type (WT) or BRAF-mutant (BRAF MUT) tumours for each histological subtype within the LMC and VisioMel test sets. SSM, superficial spreading melanoma; NM, nodular melanoma; LMM, lentigo maligna melanoma; ALM, acral lentiginous melanoma.

Histological subtypes differ in both clinical presentation and molecular profiles. For example, fewer than 20% of acral lentiginous melanoma (ALM) cases harbour BRAF mutations, compared with >60% of superficial spreading melanomas (SSMs) (42). ALM is rare in white populations but more common in non-white populations and is often diagnosed at a later stage, contributing to poorer outcomes (43). In Figure 7, all models showed 0% sensitivity and AUROC of 0.0 for ALM cases in the VisioMel external validation set. However, ALM cases were very limited (*n* = 5 BRAF-mutant cases), making it difficult to assess model performance, and the training dataset contained only 7 BRAF-mutant ALMs, likely restricting the ability of the models to learn or generalise to this subtype. For the more prevalent subtypes (SSM and NM), AUROC differences were small in the VisioMel dataset, with overlapping confidence intervals, suggesting there is no evidence of performance bias between these two subtypes.

We also evaluated model performance by anatomical site. Trunk and non-trunk melanomas exhibit distinct patterns of genetic alteration, with trunk melanomas having a higher BRAF mutational frequency (32). Although 95% confidence intervals overlapped, AUROC was generally higher for non-trunk melanomas compared with trunk melanomas (Supplementary Figures S1, S2), but this may reflect greater representation of non-trunk cases in the training dataset. No consistent patterns in performance were observed across age groups, with differences generally falling within 95% confidence intervals (Supplementary Figures S3, S4). Differences by sex were also small across most models, although reduced performance for female patients was observed for models using Phikon-v2 and Virchow2 features in the VisioMel dataset (Supplementary Figure S6). Overall, there is limited evidence of systematic performance differences across these subgroups.

## 4 Discussion

In this study, we conduct, to our knowledge, the most extensive benchmarking analysis to date of state-of-the-art pathology foundation models combined with ABMIL for image-based prediction of BRAF status in melanoma WSIs. This includes the evaluation of six foundation models across 740 WSIs, including 601 external cases. A key contribution of our work is the depth and scale of external validation: in addition to internal cross-validation, we evaluate all models on the VisioMel dataset (*n* = 523) (35), which represents one of the largest publicly available external test sets available for this task. Previous studies have typically reported performance either only on internal datasets or on substantially smaller external cohorts (ranging from 19 to 131 WSIs) (6, 8, 11, 44, 45), largely reflecting the limited availability of large, curated external datasets at the time those works were conducted. By leveraging both VisioMel and TCGA external validation sets, and by systematically comparing a larger number of foundation models than prior work, we provide a more comprehensive assessment of model generalisability and potential failure modes. Among the evaluated models, H-Optimus-1 features with ABMIL achieved the highest AUROC on the VisioMel external validation set (0.791 [95% CI 0.754–0.828]). However, because sensitivity is a key consideration for screening-oriented applications, the UNI2-h ABMIL model may be more suitable for such use cases. This model achieved the highest sensitivity on VisioMel (0.747 [95% CI 0.688–0.803]) and the second-highest sensitivity on the TCGA external dataset (0.667 [95% CI 0.526–0.805]), following the Phikon-v2 model, which was pretrained on TCGA. Despite these promising results, the sensitivity of all models was ≤0.80 (Figures 1B–H), so future studies should focus on improving model sensitivity for screening patients. In addition, CONCH-based models showed performance comparable to H-Optimus-1 and UNI2-h based models, despite CONCHv1.5 having substantially fewer parameters (307M vs. 681M for UNI2-h and 1.1B for H-Optimus-1 [Table 1)). CONCHv1.5 features also appeared less affected by dataset-specific artefacts (Figure 4D), which may be advantageous for real-world deployment.

Exploratory analysis of high attention patches and heatmaps revealed that models trained with H-Optimus-1, Phikon-v2, and UNI2-h appeared to learn high attention scores for tumour regions containing melanocytes with large, round nuclei (Figure 2). These cytological features have previously been associated with BRAF mutant tumours (6) and suggest models could be learning BRAF-correlated morphology. Yet, models appear to fail when faced with rare cellular variants, or variation in slide staining. Misclassification of spindle cell variants mirrors observations from (44), whose CLAM-based model (a variant of ABMIL) which used UNI foundation model features (the previous version of UNI2-h), misclassified a BRAF-mutant tumour with a fascicular, spindle-cell architecture—similar to the LMC case. Spindle-cell melanoma is a rare variant and likely represents an out-of-distribution morphology for these models. Rao et al. (44) suggested that, when confronted with rare variants that do not display typical BRAF-associated cytology, models may rely more on architectural patterns than on cytologic detail-highlighting a key challenge in developing robust classifiers for melanoma, a tumour type known for extreme morphological heterogeneity.

We also found that clinical factors could influence model performance, particularly ulceration status and Breslow thickness (Figures 5, 6). Prior work similarly suggests that combining clinical variables with imaging can improve predictive accuracy (6, 7). For example, (46) demonstrated that clinical variables—including age, tumour location, histologic subtype, ulceration, lymphovascular invasion, and association with a nevus—achieved an AUROC of 0.79 (95% CI: 0.74–0.84), sensitivity of 89.4% (95% CI:84.5%–93.1%), and specificity of 50.7% (95% CI: 42.2%–59.1%) for predicting BRAF status. Building on these findings, future work will explore multimodal models integrating clinical variables with foundation-model features to assess whether this improves sensitivity.

Another source of potential bias in this study is the use of different molecular assays across hospital laboratories to determine BRAF status within the LMC dataset. These assays vary in sensitivity and specificity, which can introduce noise into the ground truth labels. Consequently, this label heterogeneity may reduce model performance and introduce systematic bias. For example, low-sensitivity assays may underestimate mutation prevalence in samples with low tumour cellularity, potentially leading the model to learn spurious correlations between morphological features (e.g., cellularity) and mutation status. Furthermore, as illustrated in Figure 4, models may already capture institution-specific artifacts, which could be caused or exacerbated by ground truth labels that are themselves influenced by the assay types used across different sites.

A further limitation of our study is that we did not assess whether additional genetic alterations confound model predictions. Recent work by (47) biomarker-prediction models can be influenced by co-occurring mutations or clinical variables that correlate with the target biomarker. Consistent with this, (44) reported that several BRAF-wild-type cases misclassified as BRAF mutant carried other oncogenic alterations, such as NRAS or NF1 mutations. In our study, although we analysed performance across multiple clinical subgroups and histological subtypes and anatomical sites which are associated with different molecular profiles, we were unable to systematically account for additional genomic alterations that may contribute to model outputs. In this context, our results should be interpreted as reflecting the prediction of BRAF-correlated morphological patterns rather than exclusively BRAF-specific signals. In addition, whilst the LMC and TCGA cohorts contained a mixture of BRAF variants, the VisioMel external validation dataset consisted exclusively of V600E mutations. BRAF mutations are grouped into three classes that activate distinct downstream signalling pathways and may give rise to different cellular phenotypes. This imbalance may therefore influence model performance and limit generalisability. In particular, V600E mutations (a Class I mutation) were highly represented in the LMC (78%) and TCGA (91%) datasets, which may partially explain the strong performance observed in the VisioMel external test set. In contrast, other variants such as V601E (Class II) and V600G (Class I) were rare (e.g., only three cases in the TCGA external validation set), and additional mutation classes were insufficiently represented to robustly assess model performance across mutation subtypes. Consequently, the model may be biased toward recognising morphological features associated with V600E mutations, with uncertain performance on less common BRAF variants.

Together these findings emphasise that even large-scale foundation models remain vulnerable to out-of-distribution conditions. This is particularly evident for models using Virchow2 features. Despite Virchow2 being pretrained on the largest image corpus (approximately 3.1 million slides), including a substantial proportion of skin tissue (approximately 10%), our results indicate that pretraining scale alone does not guarantee optimal downstream performance. These observations suggest that future work may benefit from more targeted, multi-centre melanoma data collection, particularly for underrepresented groups such as ALM subtypes, thinner tumours, rare cell variants, and less common BRAF mutation classes. Additionally, training ABMIL models on multi-centre datasets may help mitigate the impact of dataset-specific artefacts (Figure 4), which could arise from variation in clinical workflows, such as differences in molecular assays across institutions.

Finally, although our work evaluates several of the most recently released foundation model architectures, providing a more up-to-date comparison than was possible in previous studies (8, 45), our analysis was limited to models that are publicly accessible to the research community. Feature extraction was performed through the TRIDENT and Patho-Bench platforms to ensure a consistent and reproducible pipeline for downstream ABMIL training (14, 15). However, there are other foundation models such as PLUTO-4 (48), which are trained on large melanoma datasets, may generate better results. Future evaluations should consider melanoma-specialised foundation models as they become available. Together, these findings indicate that while pathology foundation model features coupled with ABMIL achieve competitive performance for BRAF biomarker prediction, key limitations including inadequate sensitivity, susceptibility to rare morphologies and staining variation, and potential genomic confounding must be addressed to enable clinical deployment. Future efforts should focus on improving model robustness through multimodal integration, broader genomic characterisation, and evaluation using melanoma-focused foundation models trained on diverse, high-quality datasets.

## Data Availability

The data analyzed in this study is subject to the following licenses/restrictions: for the Leeds Melanoma Cohort, access needs to be requested, but the VisioMel and TCGA datasets are publicly available. Reasonable requests to access the LMC dataset should be directed to emily.clarke6@nhs.net.

## References

[1] Cancer Research UK. Melanoma skin cancer statistics. (2026). Available online at: https://www.cancerresearchuk.org/health-professional/cancer-statistics/statistics-by-cancer-type/melanoma-skin-cancer (Accessed March 20, 2026).

[2] Ismail RK Suijkerbuijk KP de Boer A van Dartel M Hilarius DL Pasmooij A, et al. Long-term survival of patients with advanced melanoma treated with BRAF-MEK inhibitors. Melanoma Res. (2022) 32(6):460–8. 10.1097/CMR.0000000000000832 35703270 PMC9612708

[3] Sosman JA Kim KB Schuchter L Gonzalez R Pavlick AC Weber JS, et al. Survival in BRAF V600–mutant advanced melanoma treated with vemurafenib. N Engl J Med. (2012) 366(8):707–14. 10.1056/NEJMoa1112302 22356324 PMC3724515

[4] NICE National Institute for Health and Care Excellence. Skin cancer: quality statement 5: genetic testing. (2026). Available online at: https://www.nice.org.uk/guidance/qs130/chapter/Quality-statement-5-Genetic-testing (Accessed March 20, 2026).

[5] Mistry K Jeffrey P Levell NJ Kennedy O Richardson K Craig P, et al. A national cohort study of melanoma BRAF status, testing patterns, patient and tumour characteristics, treatment and survival in England from 2016 to 2021. Br J Dermatol. (2025) 193(6):1146–54. 10.1093/bjd/ljaf351 40902091 PMC12626136

[6] Kim RH Nomikou S Coudray N Jour G Dawood Z Hong R, et al. Deep learning and pathomics analyses reveal cell nuclei as important features for mutation prediction of BRAF-mutated melanomas. J Invest Dermatol. (2022) 142(6):1650–8. 10.1016/j.jid.2021.09.034 34757067 PMC9054943

[7] Schneider L Wies C Krieghoff-Henning EI Bucher TC Utikal JS Schadendorf D, et al. Multimodal integration of image, epigenetic and clinical data to predict BRAF mutation status in melanoma. Eur J Cancer. (2023) 183:131–8. 10.1016/j.ejca.2023.01.021 36854237

[8] Albahri M Sauter D Nensa F Lodde G Livingstone E Schadendorf D, et al. A new approach combining a whole-slide foundation model and gradient boosting for predicting BRAF mutation status in dermatopathology. Comput Struct Biotechnol J. (2025) 27:2503–14. 10.1016/j.csbj.2025.06.017 40547452 PMC12182775

[9] Hernandez-Perez C Jimenez-Martin L Vilaplana V. Bridging domains in melanoma diagnostics: predicting BRAF mutations and sentinel lymph node positivity with attention-based models in histological images. In: *Proceedings of the IEEE/CVF conference on computer vision and pattern recognition (CVPR) workshops* (2024), 5132–40.

[10] Wang X Zhao J Marostica E Yuan W Jin J Zhang J, et al. A pathology foundation model for cancer diagnosis and prognosis prediction. Nature. (2024) 634(8035):970–8. 10.1038/s41586-024-07894-z 39232164 PMC12186853

[11] Wang YK Tydlitatova L Kunz JD Oakley G Chow BKB Godrich RA, et al. Screen them all: high-throughput pan-cancer genetic and phenotypic biomarker screening from H&E whole slide images. *arXiv* [Preprint]. *arXiv:2408.09554* (2025). Available online at: https://arxiv.org/abs/2408.09554 (Accessed March 20, 2026).

[12] Zimmermann E Vorontsov E Viret J Casson A Zelechowski M Shaikovski G, et al. Virchow2: scaling self-supervised mixed magnification models in pathology. *arXiv* [Preprint]. *arXiv:2408.00738* (2024). Available online at: https://arxiv.org/abs/2408.00738 (Accessed March 20, 2026).

[13] Campanella G Kumar N Nanda S Singi S Fluder E Kwan R, et al. Real-world deployment of a fine-tuned pathology foundation model for lung cancer biomarker detection. Nat Med. (2025) 31(9):3002–10. 10.1038/s41591-025-03780-x 40634781 PMC12443599

[14] Vaidya A Zhang A Jaume G Song AH Ding T Wagner SJ, et al. Molecular-driven foundation model for oncologic pathology. *arXiv* [Preprint]. *arXiv:2501.16652* (2025). Available online at: https://arxiv.org/abs/2501.16652 (Accessed March 20, 2026).

[15] Zhang A Jaume G Vaidya A Ding T Mahmood F. Accelerating data processing and benchmarking of AI models for pathology. *arXiv* [Preprint]. *arxiv:2502.06750* (2025). Available online at: https://arxiv.org/abs/2502.06750 (Accessed March 20, 2026).

[16] Bioptimus. H-Optimus-1 foundation model. *Software Model*, Bioptimus (2025). Available online at: https://huggingface.co/bioptimus/H-optimus-1 (Accessed 20 March 2026).

[17] Filiot A Jacob P Mac Kain A Saillard C. Phikon-v2, a large and public feature extractor for biomarker prediction. *arXiv* [Preprint]. *arXiv:2409.09173* (2024). Available online at: https://arxiv.org/abs/2409.09173 (Accessed March 20, 2026).

[18] Chen RJ Ding T Lu MY Williamson DF Jaume G Song AH, et al. Towards a general-purpose foundation model for computational pathology. Nat Med. (2024) 30(3):850–62. 10.1038/s41591-024-02857-3 38504018 PMC11403354

[19] Lu MY Chen B Williamson DF Chen RJ Liang I Ding T, et al. A visual-language foundation model for computational pathology. Nat Med. (2024) 30:863–74. 10.1038/s41591-024-02856-4 38504017 PMC11384335

[20] Xiang J Wang X Zhang X Xi Y Eweje F Chen Y, et al. A vision-language foundation model for precision oncology. Nature. (2025) 638:769–78. 10.1038/s41586-024-08378-w 39779851 PMC12295649

[21] Ma J Xu Y Zhou F Wang Y Jin C Guo Z, et al. PathBench: a comprehensive comparison benchmark for pathology foundation models towards precision oncology. *arXiv* [Preprint]. *arXiv:2505.20202* (2025). Available online at: https://arxiv.org/abs/2505.20202 (Accessed March 20, 2026).

[22] Kömen J de Jong ED Hense J Marienwald H Dippel J Naumann P, et al. Towards robust foundation models for digital pathology. *arXiv* [Preprint]. *arXiv:2507.17845* (2025). Available online at: https://arxiv.org/abs/2507.17845 (Accessed March 20, 2026). 10.1038/s41467-026-73923-2 PMC13260997 42277006

[23] Jaume G Doucet P Song AH Lu MY Almagro-Pérez C Wagner SJ, et al. HEST-1k: a dataset for spatial transcriptomics and histology image analysis. *arXiv* [Preprint]. *arXiv:2406.16192* (2024). Available online at: https://arxiv.org/abs/2406.16192 (Accessed March 20, 2026).

[24] Ilse M Tomczak J Welling M. Attention-based deep multiple instance learning. In: *International conference on machine learning* (PMLR) (2018), 2127–36.

[25] DeLong ER DeLong DM Clarke-Pearson DL. Comparing the areas under two or more correlated receiver operating characteristic curves: a nonparametric approach. Biometrics. (1988) 44(3):837–45. 10.2307/2531595 3203132

[26] Holm S. A simple sequentially rejective multiple test procedure. Scand J Stat. (1979) 6(2):65–70.

[27] Wilcoxon F. “Individual comparisons by ranking methods”. In: Kotz S Johnson NL, editors. Breakthroughs in Statistics: Methodology and Distribution. New York (NY): Springer (1992). p. 196–202. 10.1007/978-1-4612-4380-9_15

[28] Bauer J Büttner P Murali R Okamoto I Kolaitis NA Landi MT, et al. BRAF mutations in cutaneous melanoma are independently associated with age, anatomic site of the primary tumor, and the degree of solar elastosis at the primary tumor site. Pigment Cell Melanoma Res. (2011) 24(2):345–51. 10.1111/j.1755-148X.2011.00837.x 21324100 PMC3107974

[29] Tas F Erturk K. BRAF V600E mutation as a prognostic factor in cutaneous melanoma patients. Dermatol Ther. (2020) 33(2):e13270. 10.1111/dth.13270 32061008

[30] Heppt MV Siepmann T Engel J Schubert-Fritschle G Eckel R Mirlach L, et al. Prognostic significance of BRAF and NRAS mutations in melanoma: a German study from routine care. BMC Cancer. (2017) 17(1):536. 10.1186/s12885-017-3529-5 28797232 PMC5553744

[31] Guo W Wang H Li C. Signal pathways of melanoma and targeted therapy. Signal Transduct Targeted Ther. (2021) 6(1):424. 10.1038/s41392-021-00827-6 PMC8685279 34924562

[32] Millán-Esteban D Peña-Chilet M García-Casado Z Manrique-Silva E Requena C Bañuls J, et al. Mutational characterization of cutaneous melanoma supports divergent pathways model for melanoma development. Cancers. (2021) 13(20):5219. 10.3390/cancers13205219 34680367 PMC8533762

[33] Newton-Bishop JA Beswick S Randerson-Moor J Chang YM Affleck P Elliott F, et al. Serum 25-hydroxyvitamin D3 levels are associated with breslow thickness at presentation and survival from melanoma. J Clin Oncol. (2009) 27(32):5439–44. 10.1200/JCO.2009.22.1135 19770375 PMC2773226

[34] Newton-Bishop JA Davies JR Latheef F Randerson-Moor J Chan M Gascoyne J, et al. 25-Hydroxyvitamin D2/D3 levels and factors associated with systemic inflammation and melanoma survival in the Leeds Melanoma Cohort. Int J Cancer. (2015) 136(12):2890–9. 10.1002/ijc.29334 25403087 PMC4397121

[35] VisioMel. Data from: Visiomel challenge: predicting melanoma relapse. *VisioMel* (2023). Available online at: 10.60597/wsg0-r316 (Accessed March 20, 2026).

[36] Heath AP Ferretti V Agrawal S An M Angelakos JC Arya R, et al. The NCI genomic data commons. Nat Genet. (2021) 53(3):257–62. 10.1038/s41588-021-00791-5 33619384

[37] Xiao Y Hu Y Li B Zhang T Li Z Fu H, et al. AdaFusion: prompt-guided inference with adaptive fusion of pathology foundation models. *arXiv* [Preprint]. *arXiv:2508.05084* (2025). Available online at: https://arxiv.org/abs/2508.05084 (Accessed March 20, 2026).

[38] Bibal A Cardon R Alfter D Wilkens R Wang X François T, et al. Is attention explanation? An introduction to the debate. In: Muresan S, Nakov P, Villavicencio A, editors. *Proceedings of the 60th annual meeting of the association for computational linguistics (Volume 1: long papers)* (Dublin, Ireland: Association for Computational Linguistics) (2022). 3889–900.

[39] Godson L Alemi N Nsengimana J Cook GP Clarke EL Treanor D, et al. Immune subtyping of melanoma whole slide images using multiple instance learning. Med Image Anal. (2024) 93:103097. 10.1016/j.media.2024.103097 38325154

[40] Thiringer E Gustafsson FK Eriksson KL Rantalainen M. Scanner-induced domain shifts undermine the robustness of pathology foundation models. *arXiv* [Preprint]. *arXiv:2601.04163* (2026). Available online at: https://arxiv.org/abs/2601.04163 (Accessed March 20, 2026).

[41] Balch CM Gershenwald JE Soong S Thompson JF Atkins MB Byrd DR, et al. Final version of 2009 AJCC melanoma staging and classification. J Clin Oncol. (2009) 27(36):6199–206. 10.1200/JCO.2009.23.4799 19917835 PMC2793035

[42] Greaves WO Verma S Patel KP Davies MA Barkoh BA Galbincea JM, et al. Frequency and spectrum of BRAF mutations in a retrospective, single-institution study of 1112 cases of melanoma. J Mol Diagn. (2013) 15(2):220–6. 10.1016/j.jmoldx.2012.10.002 23273605 PMC5707183

[43] Alicea GM Rebecca VW. Un-fair skin: racial disparities in acral melanoma research. Nat Rev Cancer. (2022) 22(3):127–8. 10.1038/s41568-022-00443-8 35075287 PMC9844516

[44] Rao VR Nguyen V Phung TL Sukhadia SS. Interpreting deep learning–based prediction of the BRAF V600E mutation using diagnostic whole slide images in skin cutaneous melanoma. Am J Pathol. (2026) 196(3):716–30. 10.1016/j.ajpath.2025.11.004 41391751 PMC12975350

[45] Vorontsov E Bozkurt A Casson A Shaikovski G Zelechowski M Severson K, et al. A foundation model for clinical-grade computational pathology and rare cancers detection. Nat Med. (2024) 30(10):2924–35. 10.1038/s41591-024-03141-0 39039250 PMC11485232

[46] Dudin O Mintser O Gurianov V Kobyliak N Kaminskyi D Matvieieva A, et al. Predicting BRAF mutations in cutaneous melanoma patients using neural network analysis. J Skin Cancer. (2024) 2024(1):3690228. 10.1155/jskc/3690228 39735251 PMC11671645

[47] Dawood M Branson K Tejpar S Rajpoot N Minhas FU. Confounding factors and biases abound when predicting molecular biomarkers from histological images. Nat Biomed Eng. (2026) 2:1–15. 10.1038/s41551-026-01616-8 41772176

[48] Padigela H Nofallah S Chilaparasetti AN Han R Walker A Shen J, et al. PLUTO-4: frontier pathology foundation models. *arXiv* [Preprint]. *arxiv:2511.02826* (2025). Available online at: https://arxiv.org/abs/2511.02826 (Accessed March 20, 2026).

