# Supplementary material for: An evaluation of pathology foundation models with weakly supervised learning for BRAF mutation prediction in melanoma whole slide images
Source: Front Med Technol. 2026 Sep 3;8:1835452. doi: 10.3389/fmedt.2026.1835452 (PMC13582659; doi:10.3389/fmedt.2026.1835452)
Supplement: Supplementary file 1 [file Datasheet1.pdf]

Supplementary Material

1 SUPPLEMENTARY TABLES AND FIGURES

Model_1	Model_2	AUROC_1	AUROC_2	ΔAUROC	p-value	p_{adj}
H-Optimus-1	MUSK	0.801	0.719	0.0820	2.42×10^{-3}	0.0364*
H-Optimus-1	Virchow2	0.801	0.696	0.105	4.05×10^{-3}	0.0568
H-Optimus-1	UNI2-h	0.801	0.761	0.0405	0.0305	0.397
H-Optimus-1	CONCHv1.5	0.801	0.739	0.0621	0.0363	0.435
Virchow2	Phikon-v2	0.696	0.776	-0.0803	0.0600	0.660
UNI2-h	Virchow2	0.761	0.696	0.0650	0.0783	0.783
MUSK	Phikon-v2	0.719	0.776	-0.0569	0.0919	0.827
H-Optimus-1	Phikon-v2	0.801	0.776	0.0251	0.323	1.00
UNI2-h	CONCHv1.5	0.761	0.739	0.0216	0.452	1.00
UNI2-h	MUSK	0.761	0.719	0.0415	0.126	1.00
UNI2-h	Phikon-v2	0.761	0.776	-0.0154	0.443	1.00
CONCHv1.5	MUSK	0.739	0.719	0.0199	0.451	1.00
CONCHv1.5	Virchow2	0.739	0.696	0.0434	0.316	1.00
CONCHv1.5	Phikon-v2	0.739	0.776	-0.0369	0.297	1.00
MUSK	Virchow2	0.719	0.696	0.0235	0.534	1.00

Table S1. Pairwise DeLong test comparison of AUROC values between models,when evaluated on the LMC internal validation set. Adjusted p-values are corrected using the Holm–Bonferroni method. Adjusted p-values < 0.05 are indicated by *.

Model_1	Model_2	AUROC_1	AUROC_2	ΔAUROC	p-value	p_{adj}
Virchow2	H-Optimus-1	0.612	0.791	-0.179	3.75×10^{-15}	5.63×10^{-14}***
Virchow2	UNI2-h	0.612	0.756	-0.145	3.07×10^{-9}	4.30×10^{-8}***
H-Optimus-1	MUSK	0.791	0.702	0.0891	4.85×10^{-7}	6.30×10^{-6}***
Virchow2	CONCHv1.5	0.612	0.751	-0.139	8.07×10^{-7}	9.69×10^{-6}***
H-Optimus-1	Phikon-v2	0.791	0.721	0.0695	9.19×10^{-6}	1.01×10^{-4}***
Virchow2	Phikon-v2	0.612	0.721	-0.110	1.62×10^{-5}	1.62×10^{-4}***
Virchow2	MUSK	0.612	0.702	-0.0900	2.40×10^{-4}	0.00216**
UNI2-h	MUSK	0.756	0.702	0.0547	9.05×10^{-4}	0.00724**
CONCHv1.5	MUSK	0.751	0.702	0.0493	2.35×10^{-3}	0.0165*
H-Optimus-1	UNI2-h	0.791	0.756	0.0345	0.0102	0.0610
UNI2-h	Phikon-v2	0.756	0.721	0.0350	0.0110	0.0610
H-Optimus-1	CONCHv1.5	0.791	0.751	0.0398	0.0270	0.108
CONCHv1.5	Phikon-v2	0.751	0.721	0.0297	0.138	0.414
MUSK	Phikon-v2	0.702	0.721	-0.0196	0.313	0.627
UNI2-h	CONCHv1.5	0.756	0.751	0.00536	0.763	0.763

Table S2. Pairwise DeLong test comparison of AUROC between models, when evaluated on the VisioMel external validation set. Adjusted p-values were corrected using the Holm–Bonferroni method. Adjusted p-values < 0.05 are indicated by *, < 0.01 by ** and < 0.001 by ***.

$Model_1$	$Model_2$	$AUROC_1$	$AUROC_2$	ΔAUROC	p-value	p_{adj}
UNI2-h	Virchow2	0.787	0.632	0.155	0.0243	0.365
UNI2-h	MUSK	0.787	0.684	0.103	0.0338	0.474
CONCHv1.5	Virchow2	0.747	0.632	0.115	0.0363	0.474
UNI2-h	Phikon-v2	0.787	0.708	0.0794	0.0567	0.680
H-Optimus-1	UNI2-h	0.726	0.787	-0.0615	0.214	1.00
H-Optimus-1	CONCHv1.5	0.726	0.747	-0.0218	0.676	1.00
H-Optimus-1	MUSK	0.726	0.684	0.0417	0.522	1.00
H-Optimus-1	Virchow2	0.726	0.632	0.0933	0.181	1.00
H-Optimus-1	Phikon-v2	0.726	0.708	0.0179	0.705	1.00
UNI2-h	CONCHv1.5	0.787	0.747	0.0397	0.392	1.00
CONCHv1.5	MUSK	0.747	0.684	0.0635	0.174	1.00
CONCHv1.5	Phikon-v2	0.747	0.708	0.0397	0.453	1.00
MUSK	Virchow2	0.684	0.632	0.0516	0.334	1.00
MUSK	Phikon-v2	0.684	0.708	-0.0238	0.672	1.00
Virchow2	Phikon-v2	0.632	0.708	-0.0754	0.298	1.00

Table S3. Pairwise DeLong test comparison of AUROC between models, when evaluated on the TCGA external validation set. Adjusted p-values were corrected using the Holm–Bonferroni method.

Model 1	Model 2	Sensitivity p-value	Specificity p-value
CONCHv1.5	Phikon-v2	0.812	0.188
CONCHv1.5	Virchow2	0.875	0.625
CONCHv1.5	MUSK	1.00	0.812
H-Optimus-1	CONCHv1.5	0.125	0.438
H-Optimus-1	MUSK	0.125	0.438
H-Optimus-1	Virchow2	0.188	0.625
H-Optimus-1	UNI2-h	0.312	1.00
H-Optimus-1	Phikon-v2	0.312	0.625
MUSK	Virchow2	0.750	1.00
MUSK	Phikon-v2	1.00	0.188
UNI2-h	CONCHv1.5	0.625	0.312
UNI2-h	Phikon-v2	0.625	1.00
UNI2-h	MUSK	0.875	0.625
UNI2-h	Virchow2	1.00	0.125
Virchow2	Phikon-v2	0.625	0.312

Table S4. Pairwise comparison of model performance when evaluated on the LMC internal test set using Wilcoxon signed-rank tests. Sensitivity and specificity p-values were computed across cross-validation folds.

Model 1	Model 2	Sensitivity p-value	Specificity p-value
MUSK	Virchow2	0.0625	0.812
CONCHv1.5	Virchow2	0.125	0.625
H-Optimus-1	Virchow2	0.125	1.00
UNI2-h	Virchow2	0.125	0.312
CONCHv1.5	MUSK	0.312	0.812
Virchow2	Phikon-v2	0.312	0.438
UNI2-h	MUSK	0.438	0.625
H-Optimus-1	CONCHv1.5	0.625	0.312
H-Optimus-1	MUSK	0.625	1.00
MUSK	Phikon-v2	0.625	0.812
UNI2-h	Phikon-v2	0.625	1.00
H-Optimus-1	UNI2-h	0.625	0.625
UNI2-h	CONCHv1.5	0.812	0.812
CONCHv1.5	Phikon-v2	1.00	1.00
H-Optimus-1	Phikon-v2	1.00	0.812

Table S5. Pairwise comparison of model performance when evaluated on the VisioMel external validation set using Wilcoxon signed-rank tests. Sensitivity and specificity p-values were computed across cross-validation folds.

Model 1	Model 2	Sensitivity p-value	Specificity p-value
H-Optimus-1	MUSK	0.0625	0.875
H-Optimus-1	Virchow2	0.0625	0.0625
UNI2-h	MUSK	0.0625	0.500
UNI2-h	Virchow2	0.0625	0.125
CONCHv1.5	Phikon-v2	0.0625	0.0625
MUSK	Virchow2	0.0625	0.0625
MUSK	Phikon-v2	0.0625	0.0625
Virchow2	Phikon-v2	0.0625	0.0625
CONCHv1.5	Virchow2	0.125	0.125
H-Optimus-1	Phikon-v2	0.188	0.0625
UNI2-h	CONCHv1.5	0.188	0.500
UNI2-h	Phikon-v2	0.312	0.0625
H-Optimus-1	CONCHv1.5	0.375	0.0625
CONCHv1.5	MUSK	0.812	0.188
H-Optimus-1	UNI2-h	1.00	0.438

Table S6. Pairwise comparison of model performance when evaluated on the TCGA external validation set using Wilcoxon signed-rank tests. Sensitivity and specificity p-values were computed across cross-validation folds.

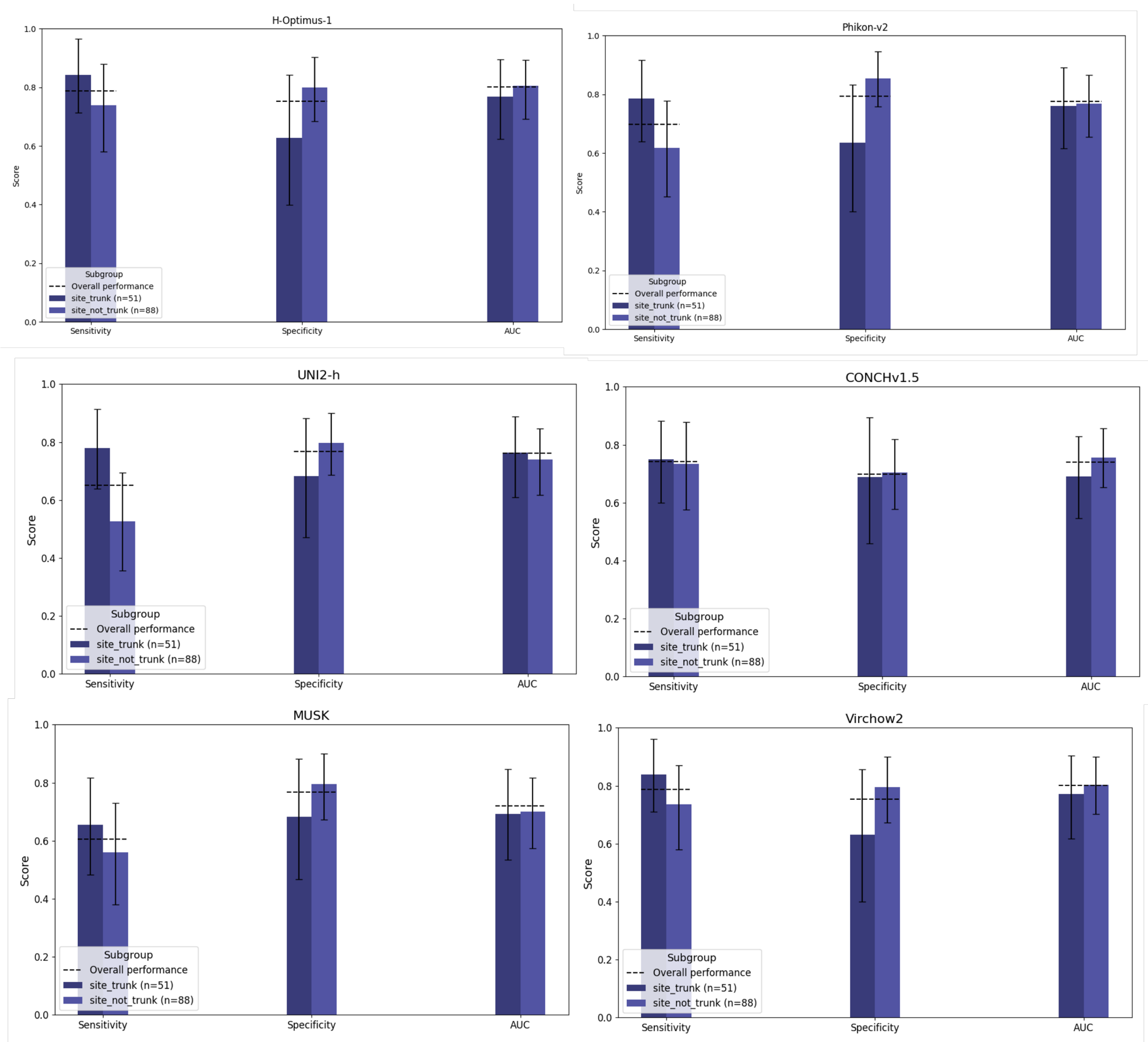

Figure S1. Performance difference based on the anatomic site of the primary tumour for the Leeds Melanoma Cohort test set (n=139).

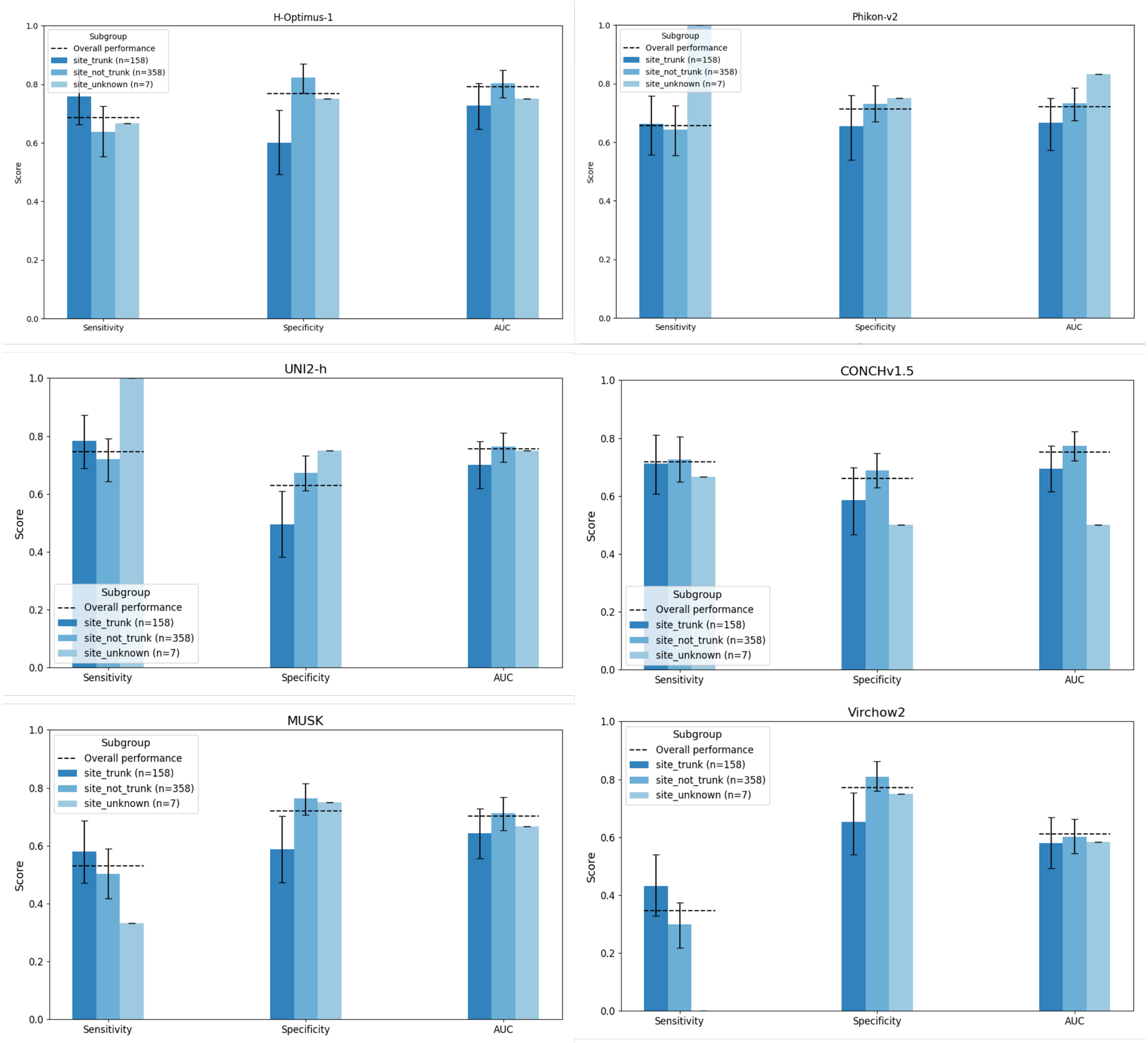

Figure S2. Performance difference based on the anatomic site of the primary tumour for the VisioMel dataset (n=523).

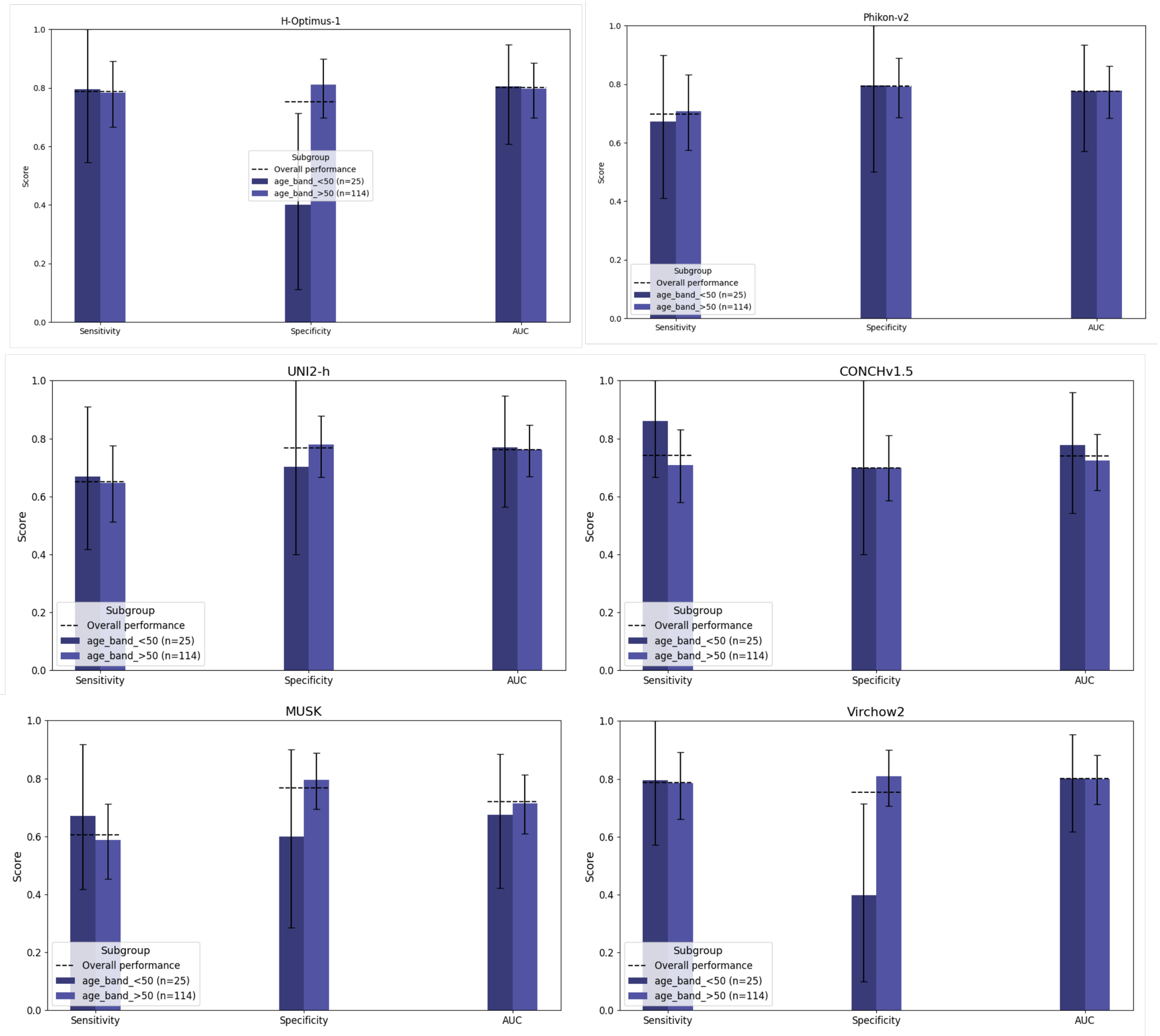

Figure S3. Performance difference based on patient age Leeds Melanoma Cohort test set (n=139).

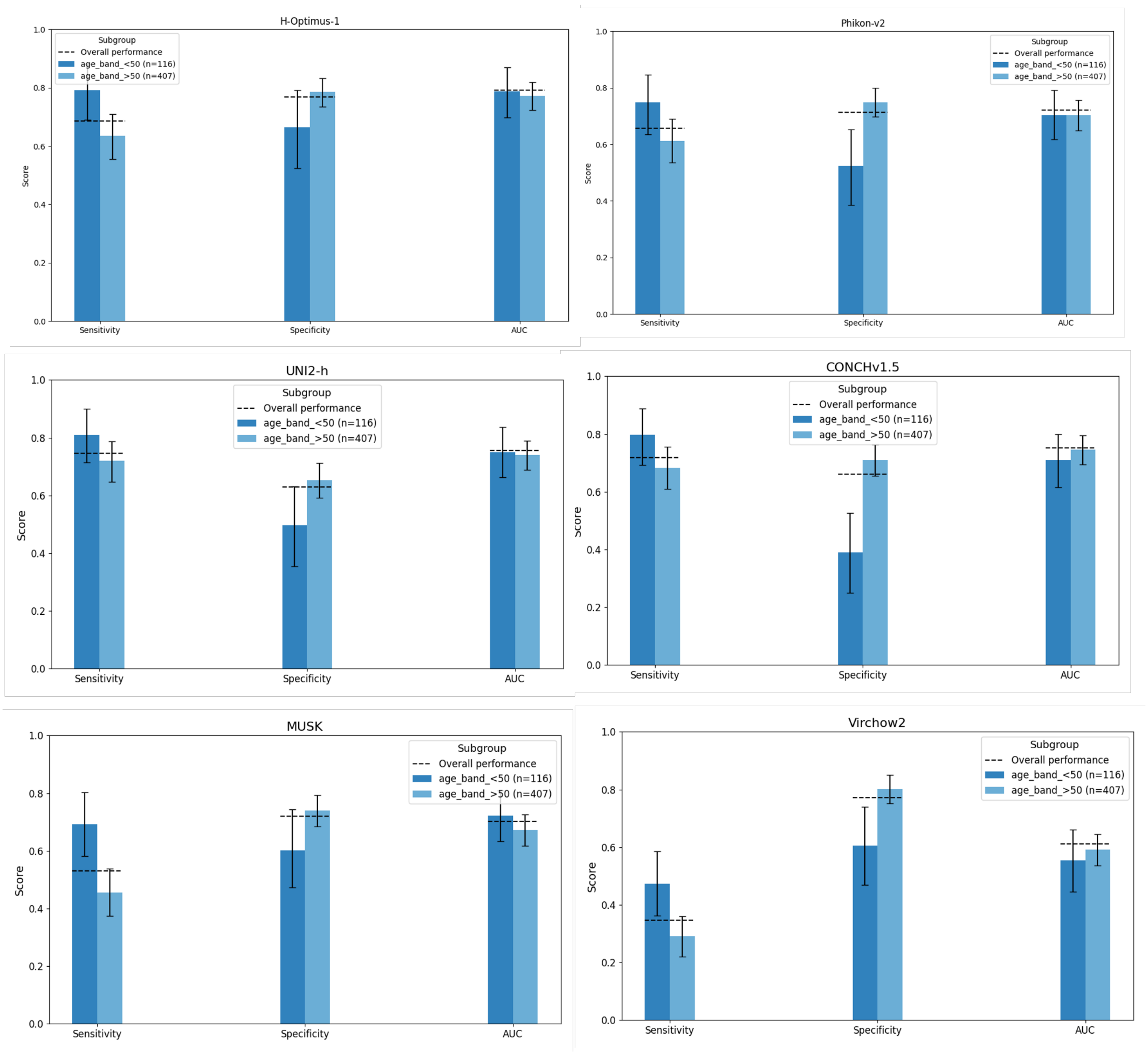

Figure S4. Performance difference based on patient age for the VisioMel dataset (n=523).

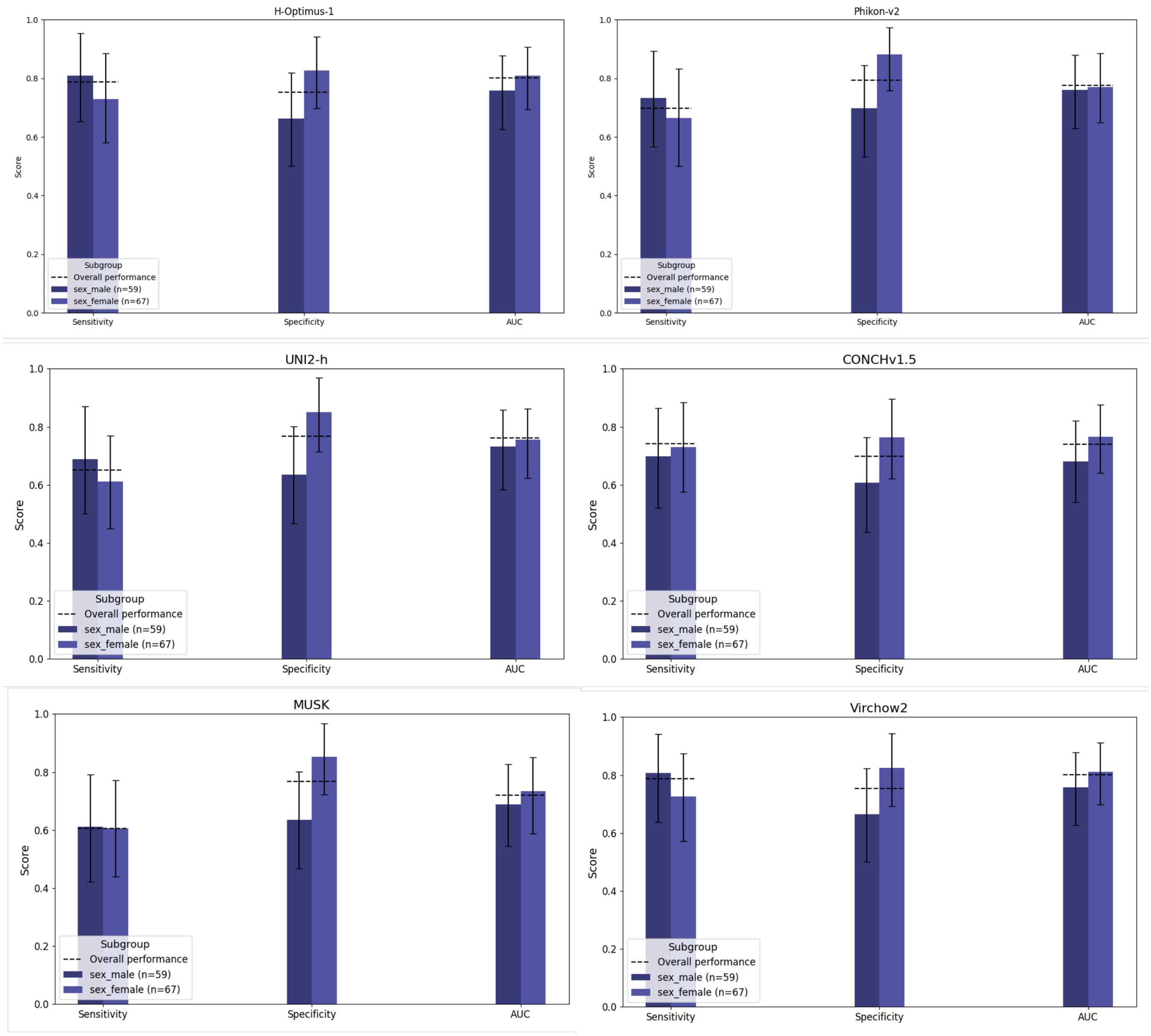

Figure S5. Performance difference based on sex for the Leeds Melanoma Cohort test set (n=139).

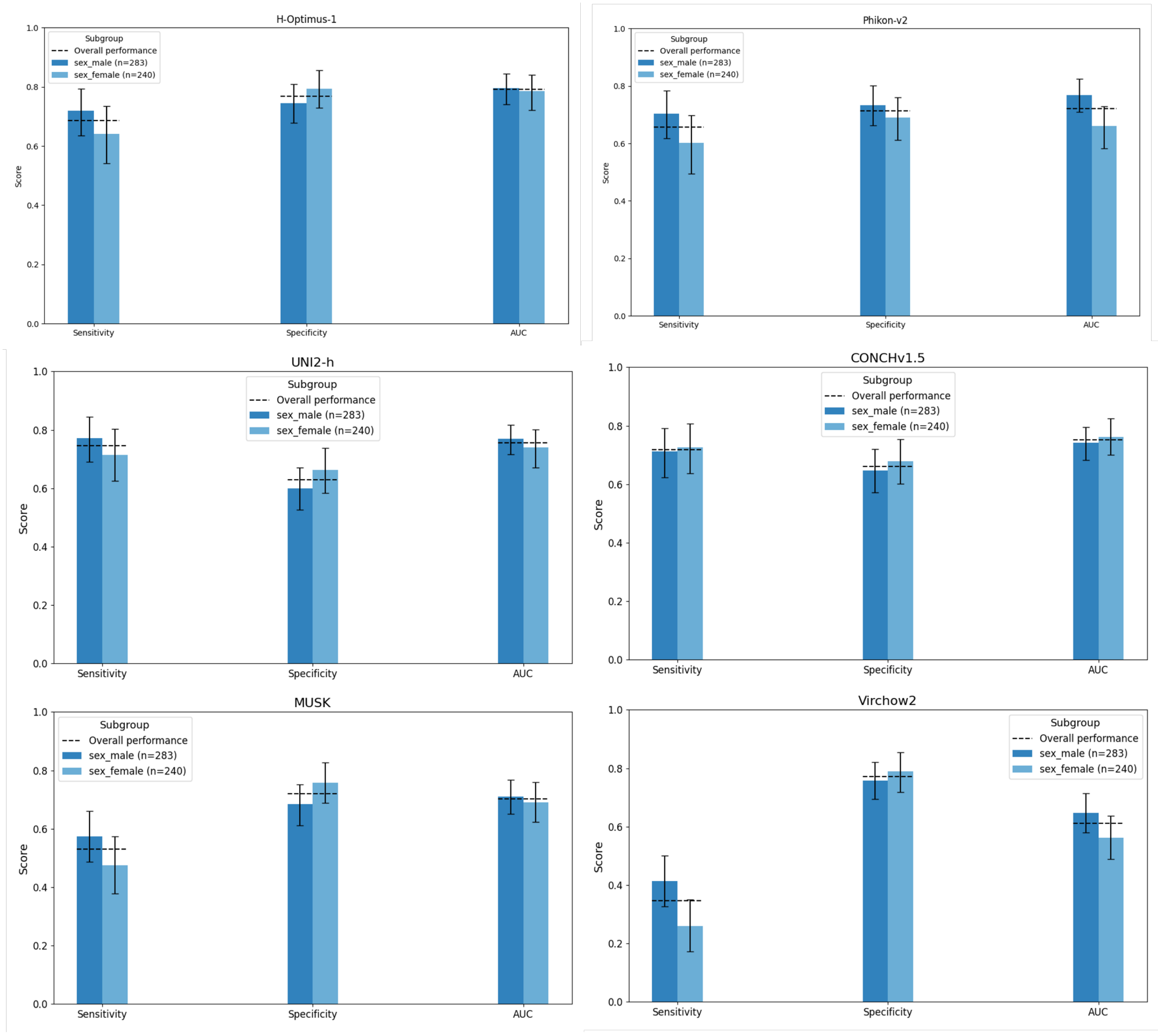

Figure S6. Performance difference based on sex for the VisioMel dataset (n=523).

Characteristic	BRAF mutant	BRAF WT
n slides (%)	472 (42.6)	637 (57.4)
n patients (%)	332 (44.6)	413 (55.4)
Age (≤50 years, %)	34.0	16.2
Sex (male, %)	38.0	43.1
**Breslow thickness category (%)**		
≤1 mm	4.5	2.9
1–2 mm	41.3	34.1
2–4 mm	35.8	38.7
>4 mm	18.1	22.8
Unclassified	0.3	1.5
**Histological subtype (%)**		
Superficial spreading	53.3	45.0
Nodular	32.5	32.2
Lentigo maligna	0.9	1.7
Acral lentiginous	2.1	4.4
Other	2.1	9.2
Unclassified	8.7	6.8
**Site of primary melanoma (%)**		
Trunk	46.4	32.7
Non-trunk	53.6	67.3
**Ulceration (%)**		
Ulcerated	29.5	35.4
Non-ulcerated	57.5	54.0
Unclassified	13.0	10.7

Table S7. Clinical and pathological characteristics of BRAF-mutant and BRAF wild-type melanoma cases within the entire Leeds Melanoma Cohort dataset. WT, wild type.

Characteristic	BRAF mutant	BRAF WT
n slides (%)	92 (44.7)	114 (55.3)
n patients (%)	66 (47.5)	73 (52.5)
Age (≤50 years, %)	22.7	13.7
Sex (male, %)	39.4	45.2
**Breslow thickness category (%)**		
≤1 mm	1.5	4.1
1–2 mm	36.4	38.4
2–4 mm	40.9	41.1
>4 mm	21.2	16.4
Unclassified	0.0	0.0
**Histological subtype (%)**		
Superficial spreading	47.0	50.7
Nodular	42.4	32.9
Lentigo maligna	1.5	4.1
Acral lentiginous	1.5	4.1
Other	1.5	5.5
Unclassified	6.1	2.7
**Site of primary melanoma (%)**		
Trunk	48.5	26.0
Non-trunk	51.5	74.0
**Ulceration (%)**		
Ulcerated	40.9	34.2
Non-ulcerated	59.1	65.8
Unclassified	0.0	0.0

Table S8. Clinical and pathological characteristics of BRAF-mutant and BRAF wild-type melanoma cases from the Leeds Melanoma Cohort test set. WT, wild type.

Characteristic	BRAF mutant (%)	BRAF WT (%)
n slides	213 (40.7)	310 (59.3)
n patients	213 (40.7)	310 (59.3)
Age (≤50 years)	31.9	15.5
Sex (male)	55.4	53.2
**Breslow thickness category**		
≤1 mm	5.6	6.5
1–2 mm	32.4	27.4
2–4 mm	30.5	32.3
>4 mm	31.0	33.9
Unclassified	0.5	0.0
**Histological subtype**		
Superficial spreading	65.3	47.7
Nodular	23.5	25.5
Lentigo maligna	0.9	5.8
Acral lentiginous	2.3	9.0
Other	4.7	6.1
Unclassified	2.3	4.2
**Site of primary melanoma**		
Trunk	39.0	24.2
Non-trunk	59.6	74.5
Unknown	1.4	1.3
**Ulceration (%)**		
Ulcerated	46.5	47.4
Non-ulcerated	50.7	48.7
Unclassified	2.8	3.9

Table S9. Clinical and pathological characteristics of BRAF-mutant and BRAF wild-type melanoma cases from the VisioMel dataset. WT, wild type.

Characteristic	BRAF mutant	BRAF WT
n slides (%)	43 (51.8)	40 (48.2)
n patients (%)	42 (53.8)	36 (46.2)
Age (% ≤50 years)	16.7	13.9
Sex (% males)	69	52.8
**Breslow thickness category (%)**		
≤1 mm	2.4	2.8
1–2 mm	4.8	0.0
2–4 mm	4.8	0.0
>4 mm	2.4	30.6
Unclassified	85.7	66.7
**Histological subtype (%)**		
Superficial spreading	2.4	0.0
Nodular	7.1	5.6
Lentigo maligna	0.0	0.0
Acral lentiginous	0.0	2.8
Other	7.1	2.8
Unclassified	83.3	88.9
**Site of primary melanoma (%)**		
Trunk	31	27.8
Non-trunk	16.7	44.4
Unclassified	52.4	27.8
**Ulceration (%)**		
Ulcerated	33.3	47.2
Non-ulcerated	7.1	22.2
Unclassified	59.5	30.6
**Race (%)**		
Asian	2.4	11.1
White	95.2	86.1
Not reported	0	2.8
Unknown	2.4	0

Table S10. Comparison of clinical and pathological characteristics between BRAF mutant and BRAF WT groups from the TCGA SKCM dataset. WT, wild type.
